# Interrater reliability estimators tested against true interrater reliabilities

**DOI:** 10.1186/s12874-022-01707-5

**Published:** 2022-08-29

**Authors:** Xinshu Zhao, Guangchao Charles Feng, Song Harris Ao, Piper Liping Liu

**Affiliations:** Department of Communication, Faculty of Social Sciences, University of Macau, Taipa, Macao

**Keywords:** Intercoder reliability, Interrater reliability, Reconstructed experiment, Cohen’s kappa, Krippendorff’s alpha

## Abstract

**Background:**

Interrater reliability, aka intercoder reliability, is defined as true agreement between raters, aka coders, without chance agreement. It is used across many disciplines including medical and health research to measure the quality of ratings, coding, diagnoses, or other observations and judgements. While numerous indices of interrater reliability are available, experts disagree on which ones are legitimate or more appropriate.

Almost all agree that percent agreement (a_o_), the oldest and the simplest index, is also the most flawed because it fails to estimate and remove chance agreement, which is produced by raters’ random rating. The experts, however, disagree on which chance estimators are legitimate or better. The experts also disagree on which of the three factors, rating category, distribution skew, or task difficulty, an index should rely on to estimate chance agreement, or which factors the known indices in fact rely on.

The most popular chance-adjusted indices, according to a functionalist view of mathematical statistics, assume that all raters conduct intentional and maximum random rating while typical raters conduct involuntary and reluctant random rating. The mismatches between the assumed and the actual rater behaviors cause the indices to rely on mistaken factors to estimate chance agreement, leading to the numerous paradoxes, abnormalities, and other misbehaviors of the indices identified by prior studies.

**Methods:**

We conducted a 4 × 8 × 3 between-subject controlled experiment with 4 subjects per cell. Each subject was a rating session with 100 pairs of rating by two raters, totaling 384 rating sessions as the experimental subjects. The experiment tested seven best-known indices of interrater reliability against the observed reliabilities and chance agreements. Impacts of the three factors, i.e., rating category, distribution skew, and task difficulty, on the indices were tested.

**Results:**

The most criticized index, percent agreement (a_o_), showed as the most accurate predictor of reliability, reporting directional *r*^2^ = .84. It was also the third best approximator, overestimating observed reliability by 13 percentage points on average. The three most acclaimed and most popular indices, Scott’s π, Cohen’s κ and Krippendorff’s α, underperformed all other indices, reporting directional *r*^2^ = .312 and underestimated reliability by 31.4 ~ 31.8 points. The newest index, Gwet’s AC_1_, emerged as the second-best predictor and the most accurate approximator. Bennett et al’s S ranked behind AC_1_, and Perreault and Leigh’s I_r_ ranked the fourth both for prediction and approximation. The reliance on category and skew and failure to rely on difficulty explain why the six chance-adjusted indices often underperformed a_o_, which they were created to outperform. The evidence corroborated the notion that the chance-adjusted indices assume intentional and maximum random rating while the raters instead exhibited involuntary and reluctant random rating.

**Conclusion:**

The authors call for more empirical studies and especially more controlled experiments to falsify or qualify this study. If the main findings are replicated and the underlying theories supported, new thinking and new indices may be needed. Index designers may need to refrain from assuming intentional and maximum random rating, and instead assume involuntary and reluctant random rating. Accordingly, the new indices may need to rely on task difficulty, rather than distribution skew or rating category, to estimate chance agreement.

**Supplementary Information:**

The online version contains supplementary material available at 10.1186/s12874-022-01707-5.

## Background

Intercoder or interrater reliability is used to measure measurement quality in many disciplines, including health and medical research [1–10]. A search of databases including Google Scholar, Scopus, and Web of Science found dozens of terms in academic literature, such as diagnostician for inter-diagnostician reliability and patient for inter-patient reliability, showing the concept’s broad reach --

annotator, arbitrator, assessor, auditor, diagnostician, doctor, editor, evaluator, examiner, grader, interpreter, interviewer, judge, monitor, observer, operator, patient, pharmacist, physician, reader, referee, reporter, researcher, respondent, scorer, screener, student, supervisor, surgeon, teacher, tester, therapist, transcriber, translator, user, voter.

Likely the earliest index is percent agreement, denoted a_o_ [9, 11]. Almost all reliability experts agree that a_o_ inflates reliability because it fails to remove chance agreement (a_c_) [2–5, 12–14]. Scores of indices have been proposed to estimate and remove a_c_. Bennett and colleagues’ S and Perreault and Leigh’s I_r_ estimate a_c_ as functions of category (C) [7, 15]. Scott’s π, Cohen’s κ and Krippendorff’s α estimate a_c_ as functions of distribution skew (s_k_) [2, 16–19]. Gwet’s AC_1_ makes a_c_ a function of both category and skew. Although many other indices are available and new indices continue to emerge, only these seven are in regular use and continue to be recommended or advocated, according to comprehensive reviews [14, 20–26].

Using derivation or simulation, statisticians discuss and debate three questions: 1) Which indices are valid or more accurate when estimating reliability or chance agreement? 2) What factors affect the indices? 3) What factors should affect the indices? Answers to Questions 2 and 3 explain the answers to Question 1 [14, 27]. Underlying the debates are five viewpoints, the first of which is widely shared by almost all experts, while the others are contested, often heatedly. The five viewpoints lead to five groups of conjectures, which we list below and leave the details to Additional file 1, Section I.2.

1. Percent agreement (a_o_) ignores chance agreement (a_c_), therefore is inflated.
2. Rating category (C) inflates S, I_r_, and AC_1_ by deflating the indices’ a_c_ estimates.
3. Distribution skew (s_k_) deflates π, κ & α by inflating the indices’ a_c_ estimates.
4. Major indices overlook task difficulty, a major factor affecting a_c_; consequently, they misestimate reliability.
5. Chance-adjusted indices, S, π, κ, α, I_r_, and AC_1_ included, assume intentional and maximum chance rating by all raters; it is under this assumption that the chance-adjusted indices share the same chance correcting formula, Eq. 1, where a_o_ is observed %-agreement, a_c_ is estimated chance agreement, and r_i_ is estimated true agreement, i.e., reliability index.

1 $${\mathbf{r}}_{\mathbf{i}}=\frac{{\mathbf{a}}_{\mathbf{o}}-{\mathbf{a}}_{\mathbf{c}}}{\mathbf{1}-{\mathbf{a}}_{\mathbf{c}}}$$

The intentional-random assumption, aka maximum-random assumption, is said to be a root cause of many known paradoxes, abnormalities, and other misbehaviors of the indices, because raters are believed to be have honestly and truthfully. Random ratings, if any, should be involuntary rather than intentional, task-dependent rather than invariably maximized [14, 21–24, 26, 28–30].

Chance agreement is a product of rater behavior, and the debates are ultimately about rater behavior [14, 31]: What behaviors are assumed by the indices’ estimations? What behaviors in fact take place? Do the assumptions match the behaviors? The debaters rely on theoretical arguments, mathematical derivation, fictitious examples, naturalistic comparisons, and Monte Carlo simulation. A systematic observation of rater behavior is needed to inform the debates over rater behavior.

This paper reports a controlled experiment that manipulated category, skew, and difficulty, and observed raters’ behavioral responses. The seven indices were tested against the observed behavior. The findings also apply to the two equivalents of a_o_, six equivalents of S, two equivalents of π, and one equivalent of κ, covering 18 indices in total, all of which had been analyzed mathematically by Zhao, Liu and Deng [14].

## Methods

### Reconstructed experiment with golden standard

#### Reconstructed experiment on real data (REORD)

We conducted a 4 × 8 × 3 between-subject controlled experiment with 4 subjects per cell. Here the term “subject” refers to the unit of analysis of a study, such as a participating patient in an experiment on the effectiveness of a new drug. A “subject” in this study, however, was a rating session with 100 pairs of rating by two raters. As 4 × 8 × 3 × 4 = 384, this study was based on 384 rating sessions, i.e., subjects. The three manipulated factors included four levels of category (C = 2,4,6,8), eight levels of difficulty (d_f_ ranges 0 ~ 1, 0 for the least and 1 for the most difficult), and three levels of skew (s_k_ = 0.5 for 50-50 distribution, 0.75 for 75-25 or 25-75 distribution, and 0.99 for 99-1 or 1-99 distribution), as summarized in Table 1.

**Table 1:** A category (C) by difficulty (d_f_) by skew (s_k_) - reconstructed experiment^a^

Across: Distribution & Skew (s_k_)		50&50 s_k_ = 0.5				25&75, 75&25 s_k_ = 0.75				1&99, 99&1 s_k_ = 0.99			
Across: Category (C)		2	4	6	8	2	4	6	8	2	4	6	8
difference in pixels (p_x_)	Difficulty d_f_ = (8-p_x_)/7												
1	=1.000	4	4	4	4	4	4	4	4	4	4	4	4
2	≈0.8571	4	4	4	4	4	4	4	4	4	4	4	4
3	≈0.7143	4	4	4	4	4	4	4	4	4	4	4	4
4	≈0.5714	4	4	4	4	4	4	4	4	4	4	4	4
5	≈0.4286	4	4	4	4	4	4	4	4	4	4	4	4
6	≈0.2857	4	4	4	4	4	4	4	4	4	4	4	4
7	≈01429	4	4	4	4	4	4	4	4	4	4	4	4
8	=0.0000	4	4	4	4	4	4	4	4	4	4	4	4

^a^Main cell entries are number of reconstructed rating sessions (subjects) in each experimental condition (cell)

Over 300 raters, registering 383 web names, from 53 Asian, European, and North American cities judged online the lengths of bars, which served as the experimental stimulus. A total of 22,290 items were rated, of which 19,900 were successfully paired, producing 9950 pairs of rating. Borrowing techniques from bootstrap [32, 33], jackknife [34], and Monte Carlo simulation [35], we sampled and resampled from the 9950 pairs to reconstruct the 384 rating sessions [36].

Thus, raters and rating were real, while rating sessions were reconstructed, making it a reconstructed experiment on real data (REORD). The Additional file 1 at the end of this manuscript (Section II) provides further details and rationales.

#### Observed true reliability (o_ri_) and true chance agreement (o_ac_) as golden standards

The raters were instructed to judge the length of bars. The researchers determined the bar lengths through programming, therefore know with certainty which rating decision was right or wrong. As the lengths of the bars were set such that random guesses would occur only between the longest and the second longest bars, the true chance agreement (o_ac_) was twice the wrong agreement (Eq. 3, Additional file 1), and true reliability (o_ri_) was observed agreement a_o_ minus o_ac_ (Eq. 5 of Additional file 1). Thus, o_ri_ served as the golden standard, namely the observed estimand, against which the seven indices were evaluated, and o_ac_ served as the golden standard for the seven chance estimators [37]. Additional file 1 (II.3) explains our use of the term "golden standard" as opposed to "gold standard."

#### Five independent variables and 16 dependent variables

Thus, this REORD experiment features three manipulated independent variables, category I, skew (s_k_) and difficulty (d_f_) and 16 main dependent variables, which are the seven indices’ reliability and chance estimations plus the observed true reliability (o_ri_) and true chance agreement (o_ca_). As the two main estimands, o_ri_ and o_ca_ sometimes also serve as independent variables when assessing their impacts on the indices’ estimations. Tables 1 and 2 and the Additional file 1 provide more details and rationales of variable calculations.

**Table 2:** Concepts and variables

		Down: Author or Origin		Reliability (True Agreement)		Chance Agreement	
		generic for any index		r_i_		a_c_	
Dependent Variables	Index Estimation	%-Agreement (unknown author)		a_o_		ao_ac_	
		Bennett et al. (1954) [15]		S		S_ac_	
		Perreault & Leigh (1989) [7]		I_r_		Ir_ac_	
		Gwet (2002, 2008, 2010, 2012) [38–41]		AC_1_		AC_ac_	
		Scott (1955) [16]		π		π_ac_	
		Cohen (1960) [2]		κ		κ_ac_	
		Krippendorff (1970, 1980) [19, 42, 43]		α		α_ac_	
	Empirical Observation	Primary Indicator		o_ri_ observed interrater reliability		o_ac_ observed chance agreement	
		Secondary Indicator (used in calculation)		o_ar_ observed right agreement		o_ae_ observed erroneous agreement	
				a_o_ observed agreement		d_o_ observed disagreement	
Independent Variables	Denotation	C		s_k_		d_f_ or e_s_	
	Concept	Category		Distribution Skew		Difficulty or Easiness	
Other Concepts	Denotation	e_m_	m_e_	s_dm_	dr^2^	N_c_	N_d_
	Concept	error of means (mean estimation minus mean target)	mean of errors (mean of differences between estimation and target)	standard deviation of an observed target of estimation (o_ae_ o_ri_)	directional *r*^2^ (dr^2^ = r*|r|)	No. of rating sessions	No. of rating decisions within a session

#### Statistical indicators – directional *R* squared (dr^2^) and mean of errors (m_e_)

Reliability indices serve two functions. One is to evaluate measurement instruments against each other, for which an index needs to accurately predict, meaning positively and highly correlating with, true reliability. We use directional r squared (dr^2^ = r•|r|) to gauge the predictive accuracy of the seven indices and their chance estimators (Table 2 and Eq. 10 of the Additional file 1). We preferred *r*^2^ over r because *r*^2^ has a clearer and more practical interpretation, percent of the DV variance explained by the IV; *r*^2^ is also more conservative as *r*^2^ ≤ |r|. We preferred dr^2^ over *r*^2^ because dr^2^ indicates the direction of the relationship while *r*^2^ does not.

The second function of the indices is to evaluate measurement instruments against fixed benchmarks, such as 0.67 and 0.80, that some reliability authorities recommend [19, 30, 44, 45]. For this function, an index needs to approximate true reliability. We use mean of errors, m_e_, which is the indices’ deviations from the observed true reliability averaged across the 384 rating sessions, to gauge the approximating accuracy of the seven indices, denoted m_e_(r_i_) in Table 2 and Eq. 8 of the Additional file 1. With the same reasoning, we also use m_e_ to assess and compare the chance estimators of the indices, denoted m_e_(a_c_) in Table 2 and Eq. 9 of the Additional file 1.

We adopted dr^2^ > .8 as the primary benchmark and m_e_ < .02 as the secondary benchmark when evaluating the seven indices. Section V of the Additional file 1 details the calculations of and the rationales behind the benchmarks.

#### Functions of *P* values and statistical pretests

This study observes the tradition of reporting *p* < α, where α = .05, .01, or .001. We however also take a functionalist view of *p* values, striving to follow the best statistical practice [46–50]:

1. avoiding the terms containing “significance," e.g., “statistical significance,” for *p* < α;
2. considering *p* < α as a prescreen threshold, passing which allows us to assess, interpret, and compare effect size indicators on percentage scales, such as *r*^2^, dr^2^ and m_e_, with some confidence;
3. using terms such as “statistical pretest” and “statistically acknowledged” where we would have traditionally used “significance test” and “statistically significant;”
4. reserving the terms containing “significant” and “significance” for effect sizes of substantive importance.

More of our views and practices regarding the functions of *p* values may be found in our prior work [51–53].

## Results

### Reliability estimations tested against observed reliabilities

**Fig. 1:**
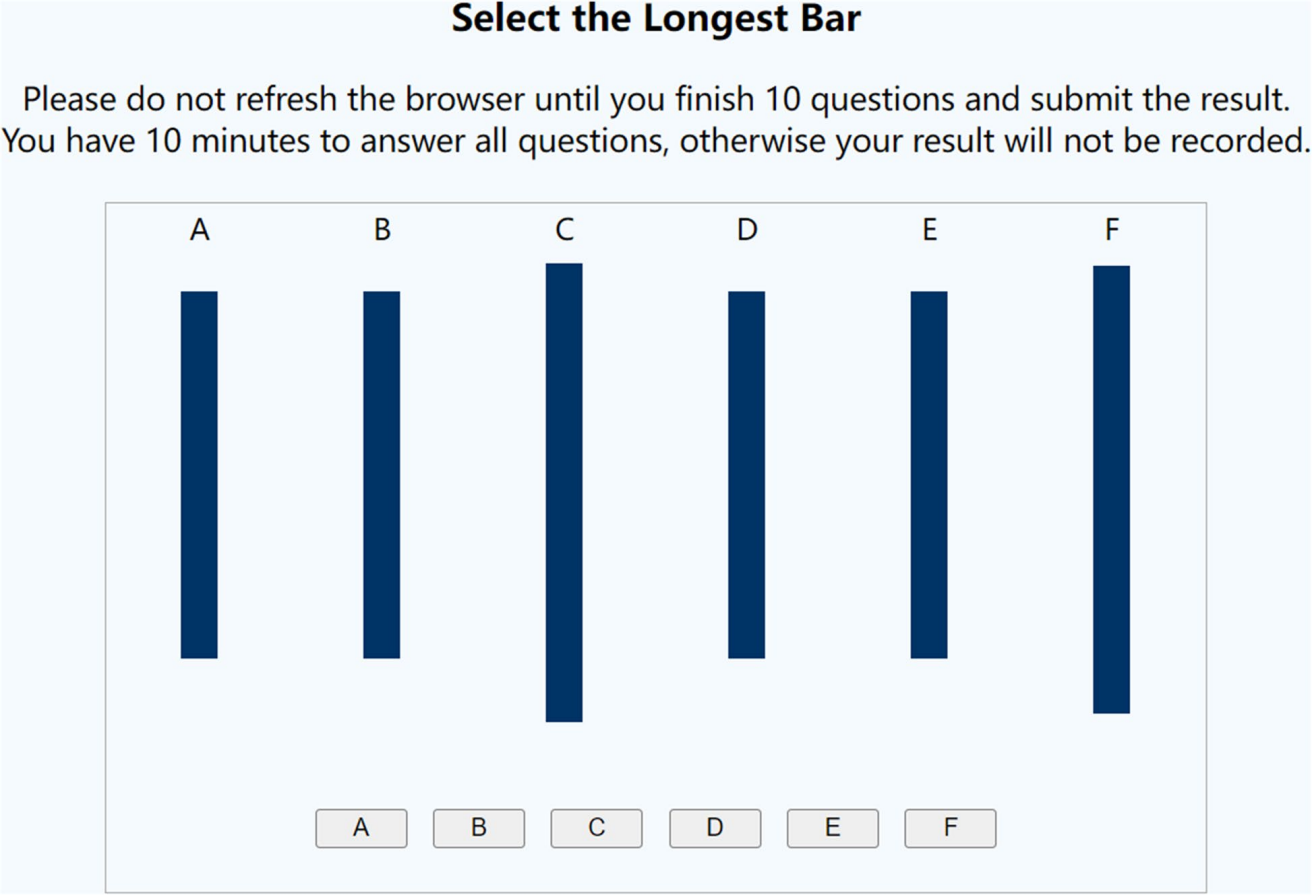
A sample screen seen by some raters (for category = 6, difficulty = 1)

Findings are summarized in Tables 3, 4, 5 and 6 and Fig. 1 and discussed in three sections. This section reports the performance of the seven indices when predicting and approximating the observed reliability. The next section analyzes the impact of the four factors on the indices’ performance. The following section discusses offset mechanism for a better understanding of the indices’ complex behavior.

**Table 3:** Effects of estimation targets, category, skew & difficulty on observed or estimated chance agreement and reliability (dr^2^)

			A.	B.	C.	D.	E.	F.	G.	H.
	1	Right: Source or Author	Observation	%-agreement	Bennett et al.	Perreault & Leigh	Gwet	Scott	Cohen	Krippendorff
Effects on Intcdr Reliability Obsv & Ests	2	Right: Obsd / Estd Interrater Reliability as Dependent Variables Down: Independent Variables	o_ri_	a_o_	S	I_r_	AC_1_	π	κ	α
	3	Observed Reliability (o_ri_)	1.00^***^	.841^***^	.691^***^	.599^***^	.721^***^	.312^***^	.312^***^	.312^***^
	4	Category (C)	.003	−.002	.175^***^	.185^***^	.123^***^	.001	.001	.001
	5	Distribution Skew (s_k_)	.000	.000	.000	−.000	.003	−.293^***^	−.292^***^	−.293^***^
	6	Difficulty (d_f_)	−.774^***^	−.778^***^	−.566^***^	−.434^***^	−.554^***^	−.389^***^	−.389^***^	−.389^***^
Effects on Chance Agrt Obsv & Ests	7	Right: Obsd / Estd. Chance Agreement as Dependent Variables Down: Independent Variables	o_ac_	ao_ac_ = 0^a^	S_ac_	Ir_ac_	AC_ac_	π_ac_	κ_ac_	α_ac_
	8	Observed Chance Agreement (o_ac_)	1.00^***^	–	.021^**^	.021^**^	.075^***^	−.151^***^	−.152^***^	−.151^***^
	9	Category (C)	−.019^**^	–	−.863^***^	−.863^***^	−.661^***^	−.013^*^	−.014^*^	−.013^*^
	10	Distribution Skew (s_k_)	−.001	–	.000	.000	−.039^***^	.437^***^	.434^***^	.437^***^
	11	Difficulty (d_f_)	.585^***^	–	.000	.000	.009	−.123^***^	−.125^***^	−.123^***^
N	12	N_c_ (number of rating sessions)	384	384	384	384	384	384	384	384
	13	N_d_ (number items within each session)	100	100	100	100	100	100	100	100

Main cell entries are directional r squared (dr^2^), which are r squared with the directional sign of r, dr^2^ = r•|r|

*: *p*<.05; **: *p*<.01; ***: *p*<.001

^a  ^As ao_ac,_ the chance estimate of a_o_, is a constant, its correlations (dr^2^) with other variables cannot be calculated

**Table 4:** Mean of errors (m_e_) / distance between index estimations and targets of estimation

			A.	B.	C.	D.	E.	F.	G.
	1	Author or Source	%-agreement	Bennett et al.	Perreault & Leigh	Gwet	Scott	Cohen	Krippendorff
Interrater Reliability	2	Interrater Reliability Estimator	a_o_	S	I_r_	AC_1_	π	κ	α
	3	m_e_ (r_i_) = mean (|r_i_-o_ri_|) (0 ≤ m_e_ ≤ 1)	.130^***^	.096^***^	.180^***^	.093^***^	.327^***^	.324^***^	.323^***^
	4	Standard Deviation of m_e_ (r_i_)	.145	.099	.148	.104	.221	.220	.220
	5	95% confidence interval of m_e_ (r_i_)	.115 ~ .144	.086 ~ .106	.164 ~ .194	.082 ~ .103	.304 ~ .349	.302 ~ .346	.301 ~ .345
Chance Agreement	6	Chance Agreement Estimator	ao_ac_	S_ac_	Ir_ac_	AC_ac_	π_ac_	κ_ac_	α_ac_
	7	m_e_ (a_c_):=mean (|a_c_-o_ac_|) (0 ≤ m_e_ ≤ 1)	.130^***^	.182^***^	.182^***^	.130^***^	.450^***^	.448^***^	.448^***^
	8	Standard Deviation of m_e_ (a_c_)	.145	.141	.141	.127	.201	.201	.202
	9	95% confidence interval of m_e_ (a_c_)	.115 ~ .144	.168 ~ .196	.168 ~ .196	.117 ~ .143	.429 ~ .470	.428 ~ .469	.427 ~ .468
**N**	10	N_c_ (number of rating sessions)	384	384	384	384	384	384	384
	11	N_d_ (number items within each session)	100	100	100	100	100	100	100

*: *p*<.05, **: *p*<.01, ***: *p*<.001

**Table 5:** Means and error of means (e_m_): index estimations against observations

			A.	B.	C.	D.	E.	F.	G.	H.
	1	Right: Author or Source	Observed Agreement	%-agreement	Bennett et al.	Perreault & Leigh	Gwet	Scott	Cohen	Krippendorff
Interrater Reliability	2	Observed or Estimated Reliability (denotation)	o_ri_	a_o_	S	I_r_	AC_1_	π	κ	α
	3	Observed / Estimated Interrater Reliability	.555	.685	.556	.726	.600	.237	.240	.241
	4	Standard Deviation	.248	.122	.203	.173	.192	.249	.247	.248
	5	Range (minimum~maximum)	−.20 ~ .90	.42 ~ .92	−.10 ~ .856	.0 ~ .925	−.045 ~ .912	−.177 ~ .778	−.173 ~ .778	−.17 ~ .779
	6	e_m_(r_i_) = mean(r_i_)-mean(o_ri_) (−1 ≤ e_m_ ≤ 1)	.000	.130^***^	.001	.171^***^	.044^***^	−.318^***^	−.315^***^	−.314^***^
	7	95% confidence interval	.00 ~ .00	.115 ~ .144	−.013 ~ .015	.155 ~ .186	.031 ~ .058	−.341 ~ −.295	−.338 ~ −.292	−.338 ~ −.291
Chance Agreement	8	Chance Agreement (denotation)	o_ac_	ao_ac_	S_ac_	Ir_ac_	AC_ac_	π_ac_	κ_ac_	α_ac_
	9	Observed or Estimated Chance Agreement	.130	.000	.260	.260	.173	.575	.573	.572
	10	Standard Deviation	.145	.000	.146	.146	.148	.109	.109	.110
	11	Range (minimum~maximum)	.0 ~ .72	.0 ~ .0	.125 ~ .50	.125 ~ .50	.022 ~ .50	.448 ~ .905	.447 ~ .905	.445 ~ .905
	12	e_m_(a_c_) = mean(a_c_)-mean(o_ac_) (−1 ≤ e_m_ ≤ 1)	.000	−.130^***^	.131^***^	.131^***^	.044^***^	.445^***^	.443^***^	.443^***^
	13	95% confidence interval	.00 ~ .00	−.144 ~ −.115	.111 ~ .15	.111 ~ .15	.026 ~ .061	.423 ~ .466	.422 ~ .465	.421 ~ .464
N	14	N_c_ (number of rating sessions)	38	384	384	384	384	384	384	384
	15	N_d_ (number items within each session)	100	100	100	100	100	100	100	100

*: *p*<.05, **: *p*<.01, ***: *p*<.001

**Table 6:** Effects of category, skew, and difficulty on observed chance agreement, reliability, and index estimations (average scores)

			A.	B.	C.	D.	E.	F.	G.	H.	I.	J.	K.	L.	M.	N	O	P	Q
			Reliability Observation or Estimation								Chance Agreement Observation or Estimation								
1	Author/ Source		Observed	%-Agreement	Bennett et al.	Perreault & Leigh	Gwet	Scott	Cohen	Krippen-dorff	Observed	%-Agreement	Bennett et al.	Perreault & Leigh	Gwet	Scott	Cohen	Krippen-dorff	
2	Estimator:		o_ri_	a_o_	S	I_r_	AC_1_	π	κ	α	o_ac_	ao_ac_	S_ac_	Ir_ac_	AC_ac_	π_ac_	κ_ac_	α_ac_	N_c_
3	Ground 0		**.555**	.685	.370	.608	.371	.369	370	.373	**.130**	0	.500	.500	.499	.501	.500	.498	32
4	Category (C)	2	**.537**	.701	.402	.584	.470	.230	.232	.234	**.164**	0	.500	.500	.401	.598	.597	.596	96
5		4	**.550**	.678	.571	.747	.621	.226	.230	.230	**.128**	0	.250	.250	.142	.573	.571	.571	96
6		6	**.557**	.676	.612	.777	.644	.239	.241	.242	**.119**	0	.167	.167	.087	.562	.561	.561	96
7		8	**.578**	.686	.641	.796	.664	.254	.257	.257	**.108**	0	.125	.125	.062	.564	.563	.562	96
8	Skew (s_k_)	.50	**.550**	.688	.560	.732	.592	.370	.372	.374	**.138**	0	.260	.260	.203	.501	.500	.498	128
9		.75	**.556**	.678	.547	.722	.588	.302	.304	.305	**.122**	0	.260	.260	.186	.545	.543	.543	128
10		.99	**.560**	.690	.561	.723	.619	.040	.044	.045	**.130**	0	.260	.260	.132	.678	.676	.676	128
11	Difficulty (d_f_)	.000	**.824**	.844	.782	.884	.810	.482	.484	.485	**.020**	0	.260	.260	.152	.630	.629	.628	48
12		.143	**.783**	.805	.728	.852	.761	.404	.406	.407	**.021**	0	.260	.260	.158	.616	.615	.615	48
13		.286	**.721**	.757	.659	.808	.697	.341	.343	.344	**.036**	0	.260	.260	.164	.599	.598	.600	48
14		.429	**.659**	.721	.600	.765	.643	.273	.275	.277	**.062**	0	.260	.260	.169	.591	.589	.588	48
15		.571	**.543**	.659	.518	.706	.563	.196	.199	.200	**.116**	0	.260	.260	.180	.565	.563	.563	48
16		.714	**.439**	.606	.444	.647	.495	.117	.121	.121	**.168**	0	.260	.260	.182	.548	.546	.546	48
17		.857	**.331**	.567	.387	.591	.440	.068	.071	.072	**.236**	0	.260	.260	.189	.534	.533	.532	48
18		1.00	**.142**	.523	.332	.552	.389	.018	.022	.022	**.380**	0	.260	.260	.194	.514	.512	.511	48
19		Mean	**.555**	.685	.556	.726	.600	.237	.240	.241	**.130**	0	.260	.260	.173	.575	.573	.572	384
20		N_d_	100	100	100	100	100	100	100	100	100	100	100	100	100	100	100	100	100

Overall, 2.86% of the raters’ decisions fell on the short bars (1.11, 1.93 and 5.53% respectively for four, six, and eight categories). As expected, there were fewer agreements on short bars, averaging 0.45% (0.04, 0.12, and 1.18%). These agreements showed no detectable effects on the main relations under investigation. The correlations between the manipulated variables were practically zero, confirming orthogonality, which indicates minimal confounding or multicollinearity.

#### Predicting reliability

Percent agreement, a_o_, the oldest and the most criticized index of interrater reliability, did well predicting true reliability, showing dr^2^ = .841 (Line 3, Table 3). Of the seven indices tested, a_o_ was the only one meeting the primary benchmark dr^2^ > .8 (Ineq. 11), outperforming the second best, AC_1_ (dr^2^ = .721), and the third best, S (dr^2^ = .691) by more than 10 points, although the latter two met the tentative benchmark dr^2^ > .67.

The most respected three, π, κ and α, tied as the least accurate predictor, reporting dr^2^ = .312, failing the tentative benchmark by margins. They also underperformed the next worst, I_r_, by 28.7 points (dr^2^ = .599).

The underperformances of the chance-adjusted indices, especially the popular π, κ and α, were disappointing, considering that the whole mission of the indices was to outperform a_o_. The low *r*^2^ means large predictive errors, suggesting that the three indices too often assign lower scores to more reliable instruments, and attach higher scores to less reliable ratings. They failed to differentiate reliable instruments from unreliable ones accurately and consistently.

**Fig. 2:**
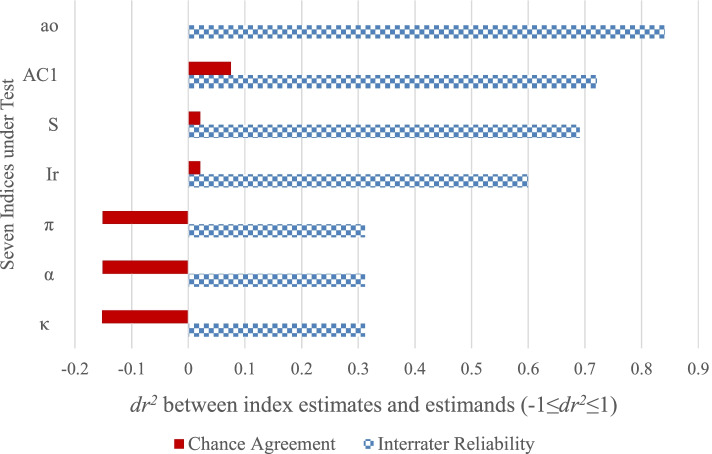
Accuracies of Interrater Reliability Indices. Notes: 1. Solid red bars are dr^2^ between estimated chance agreement & observed chance agreement. 2. Dotted blue bars are dr^2^ between estimated interrater reliability & observed interrater reliability. 3. Primary benchmark: dr^2^ > 0.8. 4. Data source: Lines 3 & 8, Table 3

Figure 2 visualizes the performances and ranks the indices by their dr^2^ scores. It is noticed, again, that κ and α ranked among the lowest while percent agreement (a_o_) ranked the highest. Figure 2 also shows a strong and positive correlation between accuracy of predicting chance agreement and accuracy of predicting interrater reliability (dr^2^ = .9768, *p* < .001), supporting a design feature of this study, which is to analyze the indices’ chance estimates for the purpose of understanding the indices.

#### Approximating reliability

A .555 average reliability (o_ri_) was observed (A3, Table 5). The seven indices’ estimation of reliability, however, ranged from .237 (π) to .726 (I_r_), indicating large approximation errors. As the experts would have predicted, percent agreement (a_o_) overestimated reliability, reporting e_m_ = .13 (B6, Table 5) and m_e_ = .13 (A3, Table 4). The error, however, was below what’s allowed by the secondary benchmark, m_e_ < .2 (Ineq. 13 of the Additional file 1). So a_o_ was the only index meeting both primary and secondary benchmarks.

Three other indices also met the m_e_ < .2 benchmark, of which two, AC_1_ (m_e_ = .093) and S (m_e_ = .096). also outperformed a_o_ (Line 3 Table 4).

The trio, π, κ and α, again underperformed all others, reporting m_e_ .323 ~ .327 (Line 8, Table 5). The errors equaled one third of the 0 ~ 1 scale, and more than doubled the errors of a_o_ (m_e_ = .130). I_r_ overestimated reliability across the board like a_o_ did (D6, Table 5), while κ, π and α underestimated across the board -- 23.7% ~ 24.1% estimated versus 55.5% observed (Line 3, Table 5).

AC_1_ and S underestimated some sessions while overestimated other sessions (Line 6, Table 5). Of AC_1_ and S, the under and over estimations offset each other to make the sizes (absolute values) of e_m_ much smaller than that of m_e_. Of the other five indices, e_m_ and m_e_ are about equal in size (Line 6, Table 5 vs Line 3, Table 4).

In part because of the offsets, AC_1_ and S produced near-zero or very small e_m_ errors (.001 and .044, respectively), much smaller than any of the other five indices did. By contrast, κ, π and α again produced the largest errors, reporting e_m_ ranging from −.318 ~ −.314, much worse than the next worst, I_r_ (e_m_ = .171, Line 6, Table 5).

#### Pi-kappa-alpha synchrony

As shown above, π, κ and α behaved like one index, despite the spirited debates on which of them is the best [10, 12, 54–57]. This pattern of π-κ-α synchrony persisted throughout the data.

### Impacts of four factors

The five viewpoints reviewed earlier discussed four factors behind reliability and/or reliability estimations. Now that we have observed rater behavior, we examine the true impacts of the four factors.

#### Conjecture group 1: chance agreement inflates a_o_

As said, a 13% chance agreement (o_ac_) and a 55.5% reliability (o_ri_) were observed, while percent agreement (a_o_) assumed 0% chance agreement and reported a 68.5% reliability, which means a 13-point overestimation (Tables 4 and 5). Conjecture 1 and the century-old beliefs were supported.

1. Chance agreement exists.
2. By completely overlooking chance agreement, a_o_ inflates the estimated reliability.
  The data from this experiment, however, adds a third point:
3. The chance agreement may not be as large as previously thought.

In this experiment, the chance agreement of a_o_ stayed below the .2 threshold, which was a main factor that allowed the predictive accuracy (*r*^2^) of a_o_ to stay above the .8 threshold. As a_o_ outperformed all six indices on the primary benchmark (*r*^2^) and outperformed four out of the six on the secondary benchmark (m_e_), an argument could be made that overestimating and misestimating chance agreement can be as counterproductive as overlooking chance agreement.

#### Conjecture group 2, category inflates S, I_r_ & AC_1_

As critics of S, I_r_ and AC_1_ would have predicted, category (C) had large and negative effects on chance estimations S_ac_, Ir_ac_ and AC_ac_, with dr^2^ ranging −.863 ~ −.661, (*p* < .001, Line 9, Table 3). Table 6 (K4 ~ K7) shows more details, e.g., S_ac_ was 50% when C = 2 but plunged to 12.5% when C = 8. The decreases appeared large compared to the 13-point average o_ac_.

Negative effects on chance estimations contribute to positive effects on reliability estimations, as shown in the dr^2^ ranging .599 ~ .721 (*p* < .001, Line 3, Table 3). S jumped from 40.2% when C = 2 to 64.1% when C = 8 (C4 ~ C7, Table 6). The effect (difference) of 23.9 points is large compared with the 55.5-point average o_ri_. In contrast, category effects on the targets of estimations, o_ri_ and o_ac_, were tiny. Coefficients dr^2^ were respectively .003 (*p* ≥ .05) and − .019 (*p* < .01) (A4 and A9, Table 3, See Table 6, Lines 4 ~ 7, for more details).

These results support the classic theory that S and equivalents underestimate chance agreement when categories exceed two, even when additional categories are largely empty.

The tables also show that I_r_ and AC_1_ relied on category in the same fashion that S did and shared the same deficiency. The differences between the category effect on S, I_r_ or AC_1_ estimation and the category effect on observed reliability all passed the *p* < .001 pretest. At the meantime, category showed minimal effects (dr^2^ ≈ .001, p ≥ .05) on π, κ and α, as their authors intended (Line 4, Table 3).

#### Conjecture group 3: skew depresses κ, π & α

As critics of κ, π & α would have predicted, skew had substantial and positive effects on chance estimators κ_ac_, π_ac_ & α_ac_, with dr^2^ ranging .434 ~ .437 (*p* < .001, Line 10, Table 3). Table 6 (Lines 8 ~ 10) shows more details, e.g., κ_ac_ was 50% when distribution was 50&50, but rose to 67.6% when distribution changed to 1&99.

The positive effects on chance estimates led to negative effects on reliability estimates. Skew effects on the three indices were all negative, with dr^2^ ranging −.293 ~ −.292 (*p* < .001, Line 5, Table 3). When distribution changed from completely even to extremely skewed, the trio’s chance agreement estimates increased from about .5 to about .68, and in parallel their reliability estimates decreased from about .37 to about .04, a drop of over 89% (Lines 8 ~ 10, Table 6). While mathematical analyses of prior studies had predicted a drop [14, 26, 58], the empirical evidence of this study showed the drastic magnitude of the drop.

In contrast to the large effects on the index estimators, skew showed minimal effect on the observed estimands, o_ri_ and o_ac_ (*p* ≥ .05 for both dr^2^, A5 & A10, Table 3), supporting the argument that chance estimates and reliability indices should not rely on skew. Each difference between the skew effect on π, κ or α estimation and the category effect on the observed estimand passes the *p* < .001 pretest.

In another contrast, skew showed practically zero effects on S, I_r_ or their chance estimates, and a small negative effect on AC_ac_ (dr^2^ = −.039, *p* < .001, Lines 5 & 10, Table 3). So I_r_ avoided the skew effect as its authors intended, while AC_1_ reversed the effect as its author intended, although the reversed effect was small. A long-suspected pattern was confirmed empiri–lly -- κ, π & α were dependent on skew while S, I_r_ & AC_1_ were dependent on category.

#### Conjecture group 4: indices overlook task difficulty

Difficulty showed a substantial and positive effect on o_ac_ (dr^2^ = .585, *p* < .001, A11, Table 3), and a large and negative effect on o_ri_ (dr^2^ = −.774, *p* < .001, A6). A change from extremely easy to extremely difficult decreased o_ri_ by over 68 percentage points and increased o_ac_ by nearly 36 points (Columns A and I, Table 6). These effects appear large compared with 13-point average o_ac_ and 55.5-point average o_ri_, suggesting that chance estimates and reliability indices should rely on difficulty.

In contrast, difficulty had minimal effects on S_ac_, Ir_ac_ and AC_ac_ (dr^2^ = .000 ~ .009, *p* ≥ .05, Table 3) and negative effects on κ_ac_, π_ac_ & α_ac_ (dr^2^ = −.123 or − .125, *p* < .001, Table 3; c.f. Columns I & N ~ P, Lines 11 ~ 18, Table 6), implying that the indices either failed to rely on difficulty or relied on its opposite, easiness, to estimate chance agreement. Each difference between the difficulty effect on chance estimation and the difficulty effect on observed chance agreement was statistically acknowledged at *p* < .001.

Difficulty showed weaker effects on the six chance-adjusted indices (dr^2^ = −.566 ~ −.389, Line 6, Table 3) than on the estimation target o_ri_ (dr^2^ = −.774). Each difference between the difficulty effect on reliability estimation and the difficulty effect on observed reliability was statistically acknowledged at *p* < .001.

By contrast, a_o_, showed a strong and negative correlation (dr^2^ = −.778, B6, Table 3) with difficulty. The correlation was as strong as the correlation between o_ri_ and difficulty (dr^2^ = −.774, A6), suggesting the negative correlations between the chance-adjusted indices and difficulty (dr^2^ = −.566 ~ −.389) are likely due to a_o_ embedded in the indices.

Based on derivation and simulation, Gwet concluded that the indices prior to AC_1_ had not handled difficulty properly, and AC_1_ handled it better, at least than κ [38, 59, 60]. The above findings support both claims. The near zero correlation between AC_ac_ and difficulty (dr^2^ = .009, p ≥ .05, E11, Table 3), however, suggests that AC_1_ still does not handle difficulty properly.

#### Conjecture group: indices assume intentional and maximum random rating

The most direct evidence for the behavioral assumptions behind the statistical indices should come from mathematical analysis. A 2013 study provides detailed scenarios of rater behavior assumed by each of the 22 indices analyzed [14]. Readers were invited to derive mathematical formulas from the behavioral scenarios. If a reader-derived formula matches the formula for the corresponding index, then the reader should conclude that the corresponding index indeed assumes the behavioral pattern depicted in the scenario. If, for example, a formula derived from the Kappa Scenario matches the formula for Cohen’s κ [2], it would confirm that κ indeed assumes the rater behavior depicted in the Kappa Scenario. Such class exercises, for example, have shown our students that the main chance-adjusted indices all assume that raters regularly conduct intentional and maximum random rating.

This study provided corroborating empirical evidence. The indices’ chance estimates were poorly correlated with their estimands, the observed chance agreements (Table 3, Line 8). The observed chance agreement (o_ac_) explained less than 8% of the variance in each of the category-based indices’ chance estimates, S_ac_ (2.1%), I_rac_ (2.1%), and AC_ac_ (7.5%). Although the correlations were stronger for the skew-based indices’ chance estimates, π_ac_ (− 15.1%), κ_ac_ (− 15.2%), and α_ac_ (− 15.1%), the dr^2^ coefficients were all negative, suggesting that the three indices tended to give higher estimates when the true chance agreements were lower, and give lower estimates when the true chance agreements were higher. Clearly, the index-estimated random rating and the observed raters’ random rating were completely different entities. This finding supports the argument that the chance-adjusted indices assume intentional and maximum random rating while typical raters conduct involuntary and task-dependent random rating. The mismatches between the assumptions and the observations explain the negligible or negative correlations between the estimates and the estimands.

More corroborating evidence for the maximum-random assumption came from the large overestimation of chance agreement by the six chance-adjusted indices, as shown in Line 12 of Table 5 and the right half of Table 6, which are summarized in Line 19.

The more detailed and situational evidence of the behavioral assumptions come from the influences of the four factors and the indices' offset and aggravation behaviors, which are discussed below.

#### Summarizing impacts of four factors

Each index of interrater reliability implied one or more misassumptions about chance agreement. a_o_ Overlooked chance agreement. S, I_r_ and AC_1_ inappropriately relied on category. π, κ And α inappropriately relied on skew. While difficulty had a strong and positive effect on chance agreement, all chance adjusted indices failed to rely on difficulty. π, κ and α even relied on its opposite, easiness. The misassumptions, including missed, mistaken, and contra assumptions, impeded estimation. π, κ And α fared worse in part because they entailed more and more devastating misassumptions, some of which had been mistaken as evidence of sophistications.

Recall that the main mission of the chance adjusted indices is to remove chance agreement in order to improve on percent agreement. When they mishandled the factors affecting chance agreement, they misestimated chance agreement, thereby misestimated reliability. Misassumptions about the four factors are keys to understanding the indices’ underperformance.

For more detailed understandings, we discuss below the offsetting mechanism, which interacts with the assumptions and misassumptions of the indices to define the indices’ behavior.

### Offsets in reliability estimation

Puzzles may arise if one peruses Tables 3, 4, 5 and 6, five of which discussed below.

#### Puzzle 1

Each chance-adjusted index relied on a wrong factor, skew or category, to estimate chance agreement; none of them relied on the right factor, difficulty. How come some approximated chance agreement far better than the others (Line 12 of Table 5 and Line 7 of Table 4)?

#### Puzzle 2

Chance estimators barely measured the observed chance agreement o_ac_; somer even measured anti o_ac_ (C8 ~ H8 of Table 3). Given the miserable performances of the chance estimations, how come the reliability estimations were all positively and sometimes substantially correlated with the observed reliability (C3 ~ H3)?

#### Puzzle 3

Assuming a negative relation between chance agreement and reliability, one might expect that an over estimation of chance agreement leads to an under estimation of reliability. How come S overestimated chance agreement by 100% (o_ac_ = .130 compared to S_ac_ = .260, Line 9, Table 5) while also approximated reliability almost perfectly (S = .556, compared to o_ri_ = .555, Line 3, Table 5)?

#### Puzzle 4

Continued from Puzzle 3, how come AC_1_ overestimated chance agreement (e_m_ = .044, Line 12, Table 5) while also overestimated reliability (e_m_ = .044, Line 6, Table 5)?

More generally, how come across-the-board overestimations of chance agreement did not translate into across-the-board underestimations of reliability (Line 12 vs Line 6, Table 5)?

#### Puzzle 5

Continued from Puzzles 3 & 4, how come I_r_ overestimated chance agreement more than AC_1_ did (Ir_ac_ = .131 vs AC_ac_ = .044, Line 12, Table 5), while also overestimated reliability more than AC_1_ did (Ir = .171 vs AC_1_ = .044, Line 6, Table 5)?

The puzzles can be explained in part by offsets, including partial offset, over offset, and counter offset, i.e., aggravation, imbedded in the reliability formulas, some of which discussed below.

#### Category offset, skew aggravation, and skew offset

To understand Puzzle 1, first recall that, under intentional-and-maximum-random assumption, chance-adjusted indices tend to overestimate chance agreement [9, 14, 29, 44, 45, 61–63]. In this experiment, the overestimations ranged from 4.4 percentage points by AC_1_ to 44.5 points by Scott’s π, all statistically acknowledged (*p* < .001, Line 12, Table 5).

To explain Puzzle 1, we note that the category-based indices assumed that larger number of categories decreased chance agreement (C9 ~ E9, Table 3), which offset the general overestimation. The skew-based indices assumed that higher skew increased chance agreement (F10 ~ H10), which aggravated the general overestimation. AC_1_ assumed both, that is, category and skew both decreased chance agreement (E10), thereby it offset the overestimation even more than the other two category-based indices.

To illustrate the point, we follow the textbook tradition of starting from ground zero, which features two raters, two categories, and 50&50% distribution. Here, and only here, all major indices gave about the same estimates, a_c_ ≈ 0.5 (K2 ~ P2, Table 6). Under intentional-and-maximum-random assumption, two raters draw from marbles, half with one color and half another color; they rate randomly if the colors match, and honestly if mismatch [9, 14, 29, 44, 45]. Task difficulty is not a factor in this view of rater behavior.

In actual rating, however, a_c_ = 0.5 could occur only if the task is extremely difficult. In our experiment, even the most difficult (d_f_ = 1 for 1-pixel difference) condition did not reach that theoretical maximum, reporting an o_ac_ = .38 (I18, Table 6). The less difficult sessions reported significantly smaller o_ac_, averaging 0.13 across all levels of difficulty. This means a 37-point initial overestimation at the ground zero by each chance-adjusted index (e_m_ = .5-.13 = .37).

When category increased from ground zero, S_ac_, Ir_ac_ and AC_ac_ decreased quickly under the category assumption (Columns K ~ M, Row 4 ~ 7, Table 6). While the assumption was unjustified given the small change in o_ac_ (I4 ~ I7), the decrease partially offset the 37-point overestimation, making S_ac_, Ir_ac_ and AC_ac_ less inaccurate. By contrast, κ_ac,_ π_ac_ & α_ac_ rejected the category assumption to remain unchanged (Columns N ~ P), hence did not benefit from the partial offset. Thus, S_ac_, Ir_ac_ & AC_ac_ became less inaccurate than κ_ac,_ π_ac_ & α_ac_.

Now return to ground zero, then increase skew. Under the skew assumption, κ_ac,_ π_ac_ & α_ac_ increased with skew (Columns N ~ P, Row 8 ~ 10, Table 6). While the assumption was unjustified given the small change in o_ac_ (I8 ~ I10), the increase further aggravated the 37-point overestimation, making κ_ac,_ π_ac_ & α_ac_ even more inaccurate. By contrast, S_ac_ and Ir_ac_ rejected the skew assumption to remain unchanged (K ~ L, 8 ~ 10), hence did not suffer from the aggravation. Thus, κ_ac,_ π_ac_ & α_ac_ became even more inaccurate than S_ac_ & Ir_ac_.

Rather than accepting or rejecting the skew assumption, AC_ac_ reversed it, by assuming that skew reduced a_c_ (M8 ~ M10). While the assumption also mismatched the observed skew effects (I8 ~ I10), the decrease further reduced the once 37-point overestimation. Here two unjustified assumptions, category and reversed skew, joined hands to partially offset another unjustified assumption, intentional and maximum random. Thus, AC_ac_ became even less inaccurate than S_ac_ & Ir_ac_, hence the least inaccurate of the six. As the effect of intentional-and-maximum-random assumption was stronger than the other two effects combined, a net effect was that even ACac still overestimated chance agreement.

There were other under-offsets, over-offsets, and counter-offsets, i.e., aggravations, some of which discussed below. Behind multifarious offsets were multifarious assumptions about rater behaviors, which fought or allied with each other or stayed neutral to produce the multifarious outcomes. Two wrongs sometimes made one right, sometimes half right, and often three, four, or more wrongs.

#### Chance-removal offset

To understand Puzzle 2, recall that, assuming intentional and maximum random rating, index designers wanted to remove the maximum amount of chance agreement from all considerations, which requires to remove a_c_ not only from percent agreement (a_o_), but also from the realm of consideration [9, 14, 23, 24, 29, 44, 45]. Accordingly, a_c_ is subtracted twice in Eq. 1, first from a_o_ in the numerator, and second from 1 in the denominator, which represents 100% of the realm of consideration. Two offsets occurred as a result. First, a_c_ offsets a_o_ in the numerator. Second, a_c_ in the denominator offsets its own impact in the numerator. As the self-offsets weaken a_c_’s effects, a_o_ dominates Eq. 1, the indices’ estimation of reliability. That explains Puzzle 2: the weak or negative a_c_–o_ac_ correlations exerted weaker effects than the strong and positive a_o_-o_ri_ correlation.

The weaker effects still hinder. The chance estimators not only failed to fulfill their prescribed mission of improving on percent agreement, but the estimators worked against the mission. Consequently, all six indices underperformed percent agreement when predicting observed true chance agreement. Ironically, it was the supposedly “most primitive” and “flawed” percent agreement (a_o_) that worked inside the indices to keep them from performing and looking even worse ([2] p38, [12] p80).

The offsets also help to explain Puzzle 3. While S overestimated chance agreement by 13.1 points (Line 12, Table 5) on average, the chance-removal offset helped to bring down the scalar error of reliability estimation to 9.6 points (Line 3, Table 4). This across-session error contains over- and under-estimations of individual sessions, which offset each other during averaging to reduce the vector error to near zero (e_m_ = .001, Line 6, Table 5. See also the discussion of aggregation bias earlier).

By setting estimated reliability (r_i_ in Eq. 1) equal to observed reliability (o_ri_ in Eq. 5 of Additional file 1), r_i_ = o_ri_, we derive a threshold (t_h_) for a_c_, which is Eq. 2:

2 $${\mathbf{t}}_{\mathbf{h}}=\frac{{\mathbf{o}}_{\mathbf{ac}}}{\mathbf{1}-{\mathbf{o}}_{\mathbf{ri}}}\kern0.5em 0\le {\mathbf{o}}_{\mathbf{ac}}\le {\mathbf{t}}_{\mathbf{h}}\le \infty$$

For any rating session, an index accurately estimated reliability when a_c_ = t_h_, underestimated when a_c_ > t_h_, and overestimated when a_c_ < t_h_. Therefore, when o_ac_ < a_c_ < t_h_, the index overestimated both the chance agreement and the reliability, explaining Puzzle 4. Across the 384 sessions, average t_h_ would be .292 if we plug o_ac_ (.13) and o_ri_ (.555) into Eq. 2. As Table 5 shows, of the six chance-adjusted indices, the three (κ, π, α) reporting a_c_ > .292 (Line 9) also underestimated reliability (Line 6), and the three (S, I_r_, AC_1_) reporting a_c_ < .292 also overestimated reliability. At the same time, all six overestimated chance agreement (Line 12). Due to the chance-removal offset, it is possible and possibly common for some category-based indices to overestimate both chance agreement and reliability.

A previously undocumented paradox emerges from this analysis (Eqs. 1 and 2). An index estimates reliability accurately (r_i_ = o_ri_) only when it overestimates chance agreement (a_c_ > o_ac_), an index that estimates chance agreement accurately (a_c_ = o_ac_) inevitably underestimates reliability (r_i_ < o_ri_), except in the extreme and impractical situation when r_i_ = o_ri_ = 0. The paradox, applicable for all known chance-adjusted indices, is rooted in the chance-removal offset imposed by Eq. 1, which traces back to the intentional and maximum random assumption [14, 23, 24, 26].

#### Square-root over offset

To understand Puzzle 5, recall that Perreault and Leigh’s I_r_ adopts the chance estimator of S, Ir_ac_ = S_ac_, and takes the square root of S as the reliability estimation [7]. S ≤ I_r_, as I_r_ = S^½^ for 1 ≥ S ≥ 0 and I_r_ = 0 for − 1 ≥ S < 0. When chance agreement is overestimated, the square root operation constitutes an additional offset [14]. Due to the category-based over-offset of S, I_r_ overestimates chance agreement more than AC_1_; at the meantime, due to the square root over-offset of I_r_, I_r_ overestimates reliability more than AC_1_. The two offsets explain Puzzle 5.

A rating session in this experiment simulates a study. In practice, errors do not offset across studies, e.g., one study’s overestimation of Disease A does not offset another study’s underestimation of Disease B. We should not overemphasize the near-zero aggregated error by S shown in e_m_ or overlook the sizable individual errors by S shown in m_e_.

## Discussion

### Main findings

Of the seven indices, percent agreement (a_o_) stood out as the most accurate predictor of reliability (dr^2^ = .841, Table 3) and the third most accurate approximator (m_e_ = .130, Table 4). AC_1_, the newest and the least known, was the second-best predictor (dr^2^ = .721) and the best approximator (m_e_ = .093). S ranked behind AC_1_ for both functions (dr^2^ = .691, m_e_ = .096).

The most respected, the most often required, and the most often applied indices, π, κ and α, ranked the last for both functions (dr^2^ = .312, m_e_ = .323 ~ .327).

The indices’ underperformances appeared attributable to mismatches between the assumed and observed rater behaviors, and multifarious offsets and aggravations between the misassumptions. Percent agreement assumed zero random rating, leading to the 13-point overestimation of reliability. The other six indices assumed intentional and maximum random rating, leading to a 37-point initial overestimation of chance agreement at “ground zero” for interrater reliability (Line 3, Table 6).

Away from ground zero, S, I_r_ and AC_1_ assumed larger number of categories produced less chance agreement, which offset the initial overestimation, while π, κ and α assumed skewer distributions produced more chance agreement, which aggravated the overestimation. The opportune offsets and the austere aggravations explain the smaller approximation errors by the category-based indices than by the skew-based indices. Contrary to the assumptions, neither rating category nor distribution skew showed meaningful effects on the observed true chance agreement.

Difficulty exhibited a substantial and positive effects on chance agreement (dr^2^ = .585, *p* < .001, Table 3), while S, I_r_, and AC_1_ did not rely on difficulty to estimate chance agreement (dr^2^ = .000 ~ .009, *p* ≥ .05). Failing to rely on difficulty further explains the three indices’ underperformances in prediction. Moreover, π, κ & α relied on the opposite difficulty, easiness, to estimate chance agreement (dr^2^ = −.125 ~ −.123, *p* < .001), which further explains π, κ & α’s worse performances than S, I_r_, and AC_1_.

### What did the indices indicate?

An index indicates a certain concept. What did the seven indices indicate? Did they indicate what they purport to indicate?

Percent agreement a_o_ was the only index meeting the primary benchmark (dr^2^ > .8), thereby also meeting the competitive benchmark. By overlooking chance agreements, a_o_ overestimated reliability by 13 percentage points (e_m_ = m_e_ = .130, Tables 4 and 5). The error was within the range allowed by the secondary benchmark (m_e_ < .2). The overestimation appeared across the board, as shown in Columns A and B (Lines 4 through 18) of Table 6, which implies that researchers and reviewers may manage a_o_’s deficiency by discounting a certain amount, such as 15 points, treating a_o_-0.15 as a crude estimation of reliability. Overall, in this experiment percent agreement behaved as a good predictor and a 13-point over-approximator of interrater reliability.

The other six indices set out to outperform a_o_ by removing estimated chance agreement a_c_. Unfortunately, their a_c_ estimations failed to accurately estimate true chance agreement o_ac_. S_ac_, Ir_ac_, and AC_ac_ were slightly influenced by o_ac_ (dr^2^ = .021 ~ .075, *p* < .01 or *p* < .001, Table 3). They were instead strongly and negatively influenced by category (dr^2^ = −.863 ~ −.661, *p* < .001), suggesting they indicated fewness of category more than they indicated chance agreement. The other three chance estimators, π_ac_, κ_ac_ & α_ac_, predicted far less accurately. They indicated mostly skew (dr^2^ = .434 ~ .437),the opposite of observed chance agreement o_ac_, and easiness (Lines 8-10, Columns F-H, Table 3).

When Eq. 1 was used to remove a_c_, a_o_ offset some impact of a_c_, which also self-offset some. The offsets reduced the category and skew effects and kept the index-o_ri_ correlations positive (Line 3-5, Table 3). But still, a_c_, the unique core of each index, all impeded the reliability estimation. S_ac_, Ir_ac_ and AC_ac_ impeded less than π_ac_, κ_ac_, & α_ac_ did, allowing S, I_r_ and AC_1_ to predict reliability better than π, κ, & α did (Line 3, Table 3). But the reduced impediments were still impediments. Consequently, none of the chance-adjusted indices had a good chance of outperforming a_o_ when predicting reliability. Two indices, AC_1_ (m_e_ = .093) and S (m_e_ = .096), did outperform a_o_ (m_e_ = .13) for approximation, which was due more to opportune offsets between misassumptions, and less to removing chance agreements (Line 3, Table 4).

Consequently, no chance-adjusted index passed the primary benchmark dr^2^ > 0.8. Two, AC_1_ (.721) and S (.691), passed the threshold dr^2^ > 0.67 for tentative acceptance (Table 3). Being the best approximator, AC_1_ (m_e_ = .093) was the one meeting the competitive benchmark. AC_1_ and S were also two of the four indices meeting the secondary benchmark, m_e_ < .2 (Line 3, Table 3).

Category exerted some effects on AC_1_ (dr^2^ = .123) and S (dr^2^ = .175). Fortunately for the two indices, the category effects were much smaller than the estimand effects of o_ri_ (dr^2^ = .721 & .691). The two indices underestimated reliability when C = 2, and overestimated when C ≥ 4 (Columns A, C and E, Lines 4 ~ 7, Table 6). Overall, AC_1_ and S were acceptable predictors of interrater reliability, and under- or over-approximators when category was respectively under or over 3.

I_r_ (dr^2^ = .599, m_e_ = .18) failed the tentative benchmark for prediction but satisfied the secondary benchmark for proximity. It overestimated reliability across the board. Overall, I_r_ was a poor predictor and an 18-point over-approximator of interrater reliability. I_r_’s overestimation was worse when the number of categories was increased.

The performances of π, κ and α belong to another class. The trio’s estimation-estimand correlations (dr^2^ = .312) were far below the primary benchmark of dr^2^ > .8 or the tentative benchmark of dr^2^ > .67; and their approximation errors (m_e_ = .323 ~ .327) were far above the secondary benchmark m_e_ < .2. Furthermore, evenness (1-skew) exerted nearly as large effects on the trio (dr^2^ = .292 ~ .293, Line 5) as their estimand o_ri_ did (dr^2^ = .312), suggesting that the trio indicated distribution evenness nearly as much as they indicated interrater reliability. More even distributions raised π, κ and α nearly as effectively as higher reliability did, even though skew or evenness showed no effect on observed reliability or chance agreement.

Overall, π, κ & α were crude predictors of reliability and evenness, and 31-point under-approximators of reliability. They were crude because they showed large errors when predicting reliability (dr^2^ = .312) or evenness (dr^2^ = .292 ~ .293).

While dr^2^ (.292 ~ .293) were too low to make π, κ & α precise indicators of evenness or skew, they were too high to make the trio pure indicators of reliability. The correlation can be even more disconcerting if one considers its impact on the creation and diffusion of knowledge. Reviewers and researchers use the trio to screen measurements and manuscripts, while the trio systematically favor more even distributions, making the world appear flatter. It would be a collective version of the conservative bias, or evenness bias, except this one permeates scientific knowledge [64, 65]. By contrast, a_o_ showed none of this disparaging deficiency (dr^2^ = .000).

## Conclusion

Like most controlled experiments, this study had limited external validity. The raters made visual judgments, which did not represent all tasks. The categories stopped at eight. The short-bar categories were largely empty by design. Each session had only two raters. The list could go on. To avoid unwarranted generalization, we used past tense to describe the indices’ behaviors and their impact.

Our findings, however, have been speculated or predicted by the theoretical analyses, mathematical derivations and Monte Carlo simulations [14, 29, 59–63, 66–70]. These studies used no actual measures, specific tasks, human raters, or other specifics that may limit external validity. What some other studies lack in internal validity, this study provides. The validity of our collective knowledge is significantly strengthened by adding empirical studies based on observing rater behavior.

The indices were advertised to be “standard” and “global” for “general purpose” [12, 14, 42, 71]. Now that some reigning indices did not perform as advertised against one set of observed behavior, it is sufficient evidence that the indices are not general or global or standard. The burden is not on doubters to prove that the purported general indices always fail, but on defenders to produce good evidence that the indices generally perform.

Despite the lack of empirical evidence in support of the reigning indices, the spiral of inertia in their defense likely will continue for a while [26, 58]. In that event, the interpretation of π, κ and α may warrant more caution, and the application of a_o_ and AC_1_ may deserve more credence.

### Future research

#### Replication studies

More controlled experiments are called for to falsify or qualify the findings of and the theories behind this experiment, and to test the other reliability indices against their estimands [71–73].

#### New indices

New indices may be needed. Index designers may be more cautious about the assumptions that raters conduct intentional and maximum chance rating, or their chance rating is determined by skew or category. More thoughts may be given to the possibility that raters conduct instead involuntary and task-dependent random rating, and more weights given to task difficulty. The index designers are encouraged to assess and adjust their ideas and indices against behavioral data, including the data from this experiment, which will be made public upon publication of this manuscript.

#### REORD and behavior-based statistical methods

Mathematical statistics use a system of axioms and theorems to build tools for analyzing behavioral data. The REORD (reconstructed experiment on real data) methodology reverses the logic, using observed behavior to inform statistical methods. The application might not be limited to interrater reliability. REORD, for example, may open a new front for the studies of sensitivity and specificity measures, two practical tools often used in medical and health research. REORD may also help to investigate the empirical relationship between reliability and validity, two of the most fundamental concepts of scientific enquiry.

#### Rater expectations of prevalence or skew

The researchers in this REORD experiment told the raters nothing about the prevalence or the skew of the long and short bars. As prevalence and skew were programmed to vary randomly between trials and between rating sessions, the researchers themselves did not know about the prevalence or skew until data analysis, and the raters could not have guessed accurately. This design feature was chosen because it resembled one type of research condition, under which raters don’t know what to expect, therefore they don’t expect.

For some tasks, however, raters do expect about prevalence and skew, due to their prior experience with the same tasks or their prior exposure to second-hand information. A follow-up study may investigate the impact of such expectations on raters’ rating or the indices of reliability, sensitivity, and specificity.

#### Human vs machine raters

Expectations about distribution, prevalence, and skew can be programmed into artificial intelligence (AI) to aid automated diagnoses, judgements, scorings, evaluations, ratings, and other decisions by machines. Unlike human decisions and human expectations that are often vague and varying, machine decisions and machine expectations can be programmed to be super clear and super consistent [74, 75]. Topics of human-machine reliability and inter-machine reliability versus inter-human reliability could be fruitful and fascinating for research using REORD, and so could topics of sensitivity, specificity, and validity with human and/or machine raters.

## Supplementary Information

**Additional file 1.**

## Data Availability

The datasets used and/or analyzed during the current study are available from the corresponding author on reasonable request.
