# Supplementary material for: Interrater reliability estimators tested against true interrater reliabilities
Source: BMC Med Res Methodol. 2022 Aug 29;22:232. doi: 10.1186/s12874-022-01707-5 (PMC9426226; doi:10.1186/s12874-022-01707-5)
Supplement: Supplementary file 1 — Additional file 1. [file 12874_2022_1707_MOESM1_ESM.docx]

**Additional file 1 to “Interrater reliability estimators tested against true interrater reliabilities”**

I: Five Concepts and Five Viewpoints about Interrater Reliability

I.1. Five Concepts

I.1.1. Interrater Reliability (*r_i_*)

I.1.2. Chance Agreement (*a_c_*)

I.1.3. Category (*C*)

I.1.4. Distribution Skew (*s_k_*)

I.1.5. Difficulty (*d_f_*)

I.2. Five Viewpoints

I.2.1. Chance agreement inflates *a_o_*

I.2.2. Rating category inflates *S*, *I_r_*, and *AC_1_*

I.2.3. Distribution skew deflates π, κ & α

I.2.4. Reliability indices overlook task difficulty

I.2.5. Indices assume intentional and maximum random rating

II: Reconstructed Experiment with Golden-Standard Task

II.1. Manipulating Category (*C*)

II.2. Manipulating Difficulty (df)

II.3. Creating One-way Golden Standard

II.4. Pairing Rater Responses

II.5. Manipulating Skew (*s_k_*)

II.6. Reconstructing Rating Sessions

II.7. Reconstructed Experiment in Summary

III: Variable Measurements and Calculations

III.1. Calculating Chance Agreement (*o_ac_*)

III.2. Alternative Calculation of Observed Chance Agreement (*o_ac_*)

III.3. Calculating Observed Reliability (*o_ri_*)

IV: Statistical Indicators

IV.1. Approximating and predictive functions of reliability indices

IV.2. Proximity Measure I -- Error of Mean (*e_m_*)

IV.3. Proximity Measure II -- Mean of Errors (*m_e_*)

IV.4. Predictive Accuracy and Share of Influence -- Directional *r^2^* (*dr^2^*)

IV.5. Regression vs ANOVA

V: Benchmarks and Thresholds

V.1. Ideal index outperforms all others

V.2. Reliability over chance agreement

V.3. Prediction (*dr*^2^) over approximation (*m_e_* & *e_m_*)

V.4. *m_e_ over e_m_*

V.5. Primary Requirement

V.6. Secondary Requirement

V.7. Tentative Requirement

V.8. Competitive requirement

1. **Five Concepts and Five Viewpoints about Interrater Reliability**

Section I discusses five concepts and five viewpoints about interrater reliability to supplement the manuscript.

**I.1. Five Concepts**

Five fundamental concepts, interrater reliability (*r_i_*), chance agreement (*a_c_*), rating categories (*C*), distribution skew (*s_k_*), and task difficulty (*d_f_*), is explicated below (1). Indicators of *r_i_* and *a_c_* were measured as dependent variables in this experiment, while *C*, *s_k_* and *d_f_* were manipulated as the three independent variables.

**I.1.1. Interrater Reliability (***r****_i_*).** *Interrater reliability* (*r_i_*) refers to the true agreement between raters, aka coders, engaged in systematic and task-driven rather than random rating, aka coding. Indices of interrater reliability are meant to estimate this true agreement. As chance agreement (*a_c_*), defined below, is believed to inflate reliability estimate, many indices attempts to estimate and remove *a_c_* (2–6). All major indices, including the six examined in this study other than *%-agreement a_o_*, share Eq. 1 to remove *a_c_* and estimate *r_i_*.

|  | $\boldsymbol{r}_{\boldsymbol{i}}\mathbf{=}\frac{\boldsymbol{a}_{\boldsymbol{o}}{\mathbf{-}\boldsymbol{a}}_{\boldsymbol{c}}}{\mathbf{1}\mathbf{-}\boldsymbol{a}_{\boldsymbol{c}}}$ | ( | 1 | )  ) |
| --- | --- | --- | --- | --- |

A main objective of this study is to assess the seven indices of interrater reliability against observed true reliability (*o_ri_*). The eight measures also serve as dependent variables, on which the effects of category, skew, and difficulty are assessed and compared.

**I.1.2. Chance Agreement (*a_c_*).** This study also measured seven chance agreement (*a_c_*) variables, one chance estimate for each of the six chance-adjusted indices plus observed true chance agreement (*o_ac_*). There was an implied eighth chance indicator, by percent agreement (*a_o_*), which is, by definition, a constant at zero.

*Chance agreement* (*a_c_*) refers to the agreement produced by random rather than systematic and task-driven rating. Five indices invented their own chance estimators while *I_r_* adopted the estimator from *S*.

In addition to comparing the indices with observed reliability, it is important to also compare the indices’ chance estimates with observed chance agreement. In Equation 1, subtraction of *a_c_* in the nominator decreases *r_i_*, while the subtraction in the denominator increases *r_i_*. The varying offsetting obscures the differences between indices (7). Since the main or only difference between many indices is in chance estimators (*a_c_*), comparing *a_c_* with its estimation target (*o_ac_*) may tell us more about the inside mechanism at the core of the indices.

The seven chance estimate measures also serve as dependent variables, on which the effects of category, skew, and difficulty are assessed and compared.

**I.1.3. Category (C).** *Category* (*C*) was defined as the number of choices available to a rater on a nominal scale. For example, variable *gender* often has two categories, while *party affiliation* in U.S. may have four, democrat, republican, independent, and others.

**I.1.4. Distribution Skew (*s_k_*).** Distribution, aka base rate, frequency, marginal, or prevalence, refers to the pattern of percentage occurrences, e.g. 49% female and 51% male, or 5% unhealthy and 95% healthy (3,4,16,17,8–15). Major indices are symmetrical, centered on 50&50% distribution. Accordingly, this study folded the original distribution to create *distribution skew* (*s_k_*), which served as a main independent variable.

**I.1.5. Difficulty (*df*).** *Difficulty* (*d_f_*) represents the combination of all factors that make rating inaccurate, including 1) *task difficulty*: Some tasks are more difficult than others; 2) *rater difficulty*: Some raters are less capable, focused, or motivated than others, which increases difficulty; 3) *instrument difficulty*: Instruments are means that help raters to accomplish a task, including organization, instruction, training, and equipment. Deficient instruments increase difficulty. This study fixed instrument difficulty at the lower end by giving easily understood tasks and instructions. We manipulated task difficulty and assumed variation in rater difficulty.

**I.2. Five Viewpoints**

Five viewpoints have influenced experts’ understanding of interrater reliability. They are also the theoretical focal points of this study.

**I.2.1. Chance agreement inflates *a_o_*.** In the academic literature on interrater-interrater reliability, likely the earliest and the most widely received viewpoint is that percent agreement (*a_o_*) inflates reliability by overlooking *chance agreement* (*a_c_*). Consequently *a_o_* is considered “the most primitive,” (18 p38) “inadequate,” (19 pp187&193) and “flawed,” (20 p80) therefore “should *not* be used.” (19 p187,21–23). Removing chance agreement is the core or the stated mission of early indices, e.g., Benini’s β (24). Bennett et al’s *S* (25), Goodman & Kruskal’s λ_r_ (26) and Guttman’s ρ (27). Of these, only *S* remains in regular use today (28, 29).

**I.2.2. Rating category inflates *S, I_r_, and AC_1_*.** Another widely shared viewpoint is that *S* depends on category while it should not. Large number of *categories*, even if empty, deflates chance estimates of *S* (*S_ac_*), thereby inflates *S* (9–11,30–32). The criticism also applies to six equivalents or special cases of *S*, namely *C* (33), *G* (34,35), *k_n_* (36), *PABAK* (37), *RE* (38), and *redefined* *Pi* (39).

Perreault & Leigh took the square root of *S* to produce *I_r_* (5)*.* Gwet incorporated the entire *S* into his *AC_1_*. So category affects *I_r_* and *AC_1_* in a similar way as it affects *S*, according to mathematical analysis and simulation (7,9–11), although some consider *I_r_* “the best” (41,42,43 p.384). As *I_r_* regularly produces higher scores than other indices, its popularity has grown fast in some fields (44).

Eliminating category effect was a main justification for Scott (32) to offer π, which in turn inspired Cohen’s κ (18) and Krippendorff’s α (45,46). Not suffering from category effect is a main reason that methodologists recommend π, κ or α over alternatives (7,20).

**I.2.3. Distribution skew deflates π, κ & α.** Considered the “statistics of choice” (47 p140), κ is by far the most often used index across disciplines, followed by π and α (7,9,52,10,11,21,22,48–51).

A controversial viewpoint is that π, κ and α depends on distribution skew while they should not. The trio, critics argue, mistakenly assumes that more skewed distributions create more chance agreements. Consequently, higher or lower prevalence of a variable, e.g., disease, produces larger estimates of chance agreement, thereby deflates estimated reliability (3,4,16,17,21–23,29,36,37,50,51,5,52–60,6,8–12,15).

By contrast, *AC_1_* assumes a negative skew effect on chance agreement, while *I_r_* follows *S* to assume no skew effect.

The alleged dependence of π, κ and α on skew ignited repeated and spirited debates. Experts defended κ by reaffirming its validity, extending its application, or teaching its use (14,28,61–68). Rogot & Goldberg introduced *A_2_*, a mathematical equivalent of κ (69). Byrt and colleagues introduced *BAK* (37), and Siegel & Castellan introduced *Revised* *Κ* (70), which are two equivalents of π. Krippendorff advocated and defended α vigorously (20,31,71). Zwick recommended π over κ and *S* (29), while Hsu & Field recommended κ over π (28). Vach opined that the dependence on skew is harmless (107 p655), and Krippendorff acclaimed that the dependence is desirable and by design (31,71).

**I.2.4. Reliability indices overlook task difficulty.** An emerging viewpoint is that indices of interrater reliability should depend on *task difficulty,* but they do not. More difficult tasks induce more chance rating, therefore more chance agreements (3,4,72,73,7,9–13,40,53). Krippendorff , however, opined the opposite, that “more complex” tasks lead to “very small” chance agreement (71 p488).

**I.2.5. Indices assume intentional and maximum random rating.** Among the most fundamental hence the most forcefully debated views is that the chance-adjusted indices all assume intentional and maximum random rating by conspiring raters, which include all raters for all ratings, all the time (7,73–79). The raters, according to this assumption, agree *a priori* to do the following -

1) To “rate” at the commands of randomization devices, e.g., randomly thrown coins, rolled dice, or drawn marbles, virtual or actual, without looking at the subjects under rating,

2) To rate truthfully *only* when the randomization devices disagree with each other, therefore rendering no consistent command for raters to follow.

Krippendorff rejected this view regarding Krippendorff’s α, and characterized the discussion as “strange, almost conspiratorial uses of language.” (71).

Bipolar all-or-nothing assumptions were detected hidden in the indices. Percent agreement assumes absolutely no random rating, while the chance-adjusted indices assume intentional and maximum random rating. The latter group assume that raters draw virtual or actual marbles before any “rating;” they “rate” by the order of the marbles whenever the marbles agree to give a consistent order; they rate honestly only when the marbles disagree with each other thereby giving no consistent order (7,9–12,72,73,80).

Different indices assume different ways that raters arrange the virtual or actual marbles for the random drawing and rating. *S*, *I_r_* and *AC_1_* assume that raters arrange the marbles evenly across color types that are matched with rating categories, causing the triad's dependence on rating category. π, κ And α assume that raters match the distribution of marble colors to the pre-determined but post-reported target distribution, causing the trio’s dependence on target distribution and skew. As said, Krippendorff denied that α makes such assumptions (71,81–83).

The key questions, therefore, are about rater behavior. What behaviors are assumed? What behaviors take place? Do the assumptions match the behaviors? Reliability researchers rely on theoretical arguments, mathematical derivation, fictitious examples, naturalistic comparisons, and Monte Carlo simulation. A systematic observation of rater behavior is needed to inform the debates over rater behavior.

This paper reports a controlled experiment that manipulated category, skew, and difficulty, and observed raters’ behavioral responses. Seven indices of interrater reliability were tested against the observed behavior. The findings also apply to the two equivalents of *a_o_*, six equivalents of *S*, two equivalents of π, and one equivalent of κ, covering 18 indices in total.

1. **Reconstructed Experiment with Golden-Standard Task**

Section II details the design and the execution of the reconstructed experiment that provided the main empirical evidence for this study.

We programmed a website that asked raters to identify the longest bar from several bars (Figure 1 in the maintext). Two of the independent variables, category, and difficulty, were manipulated by programming the website.

**II.1. Manipulating Category (*C*).** *Category* (*C*) was manipulated by giving raters two, four, six or eight bars to choose from. Thus, *C* had four values, 2, 4, 6 and 8.

**II.2. Manipulating Difficulty (*d_f_*).** *Task* *difficulty* (*d_f_*) was manipulated by varying the differences between two longest bars. The differences ranged from one pixel, the smallest controllable element on a computer screen, to eight pixels, which were clear to nearly everyone. The variable *d_f_* was linearly transformed to a 0~1 scale where 1 represents the most difficult.

The two longest bars (*long bars*) were 200 pixels long plus or minus 0~4 pixels for the manipulation of difficulty. The lateral distance between long bars was fixed at 150 pixels to minimize distance effect.

We confined the main competition between the long bars. Few raters chose the short bars as they were clearly shorter, which made this experiment very close to Scott's empty-category assumption and minimized the correlation between *category* and *difficulty* (32).

**II.3. Creating One-way Golden Standard.** A *golden standard* is a consensus criterion under which judgments can be made with certainty. Reliability indices are standards to evaluate instruments. Now that we are to evaluate the standards, a golden standard would be helpful if available. The longest-bar task provides such a golden standard. Through programming codes, we the researchers always know with certainty which bar was the longest, and whether each rating decision was right or wrong, based on which chance agreement and true reliability can be calculated and analyzed. We use “golden standard” as a stronger term than “gold standard.” The latter term was borrowed by Rudd in 1979 from economics where it referred to the value of gold as a monetary standard (84,85).

So that variables vary, the golden standard needs to be equipped with a one-way mirror that is always crystal clear to researchers, but variably clear to participants. The longest-bar task also provides this figurative or virtual mirror, as the task was designed such that raters sometimes knew with near certainty, but sometimes did not, thereby they had opportunities to rate randomly and agree by chance.

**II.4. Pairing Rater Responses**. Each rater rated 10 items per period and was given summary statistics of right and wrong at the end of each period. The task was made to resemble an online game or IQ test to maintain raters’ attention and focus. Items per period were limited to 10 to reduce clutter effect (86,87). Number of bars, level of difficulty, and the location of long bars were randomly rotated to minimize the effects of learning, fatigue, boredom, serial position, rater idiosyncrasies, and other possible confounders (87–91).

The same 10 items were rated again in the same order by the next rater available. After completing 10 items, a rater may choose to rate 10 more. He or she might be given 10 unpaired items rated by another rater, or 10 new items if all rated items had been paired. The process repeated until the end of data collection.

The data collection took place in a three-month period. Students, teachers, researchers, technicians, managers, office workers and other professionals from 15 colleges and two research firms in America, China mainland, Hong Kong, Macau and Singapore participated as a part of their class exercises, professional training, or work assignments. They registered 383 web names and logged on from 53 Asian, European and North American cities. They rated a total of 22,290 items, of which 19,900 were successfully paired, producing 9,950 paired responses, from which we sampled and resampled to reconstruct 384 rating sessions to form a between-subject (session) experiment that we report below.

**II.5. Manipulating *Skew* (*s_k_*).** As the longest bar is either at the left or right side of the second longest bar, we defined *distribution* as the left-and-right percentage. For example, when 1% of the rated screens had the longest bar at the left, the distribution is denoted 1&99. Five levels were chosen: 1&99, 25&75, 50&50, 75&25, and 99&1, the last of which represented 99% left & 1% right. 0&100 and 100&0 were omitted as π, κ and α would be undefined.

It is skew, but not the unfolded distribution, that’s expected to affect the indices (7,12,15,16,36). Therefore, *skew* (*s_k_*) was operationalized as *distribution folded in the middle*. 1&99 and 99&1 were both assigned *s_k_*=0.99, for the highest skew. 50&50 was assigned *s_k_*=0.5 for the lowest skew, and 25&75 and 75&25 were both assigned *s_k_*=0.75 for moderate skew. Variable *skew* (*s_k_*) ranged 0.5~0.99.

**II.6. Reconstructing Rating Sessions.** To reconstruct the first rating session, we randomly sampled without replacement 100 paired rating responses (*N_t_*=100) requiring two *categories* (*C*=2), lowest *difficulty* (*d_f_*=0), and highest *skew* (*s_k_*=.99). After recording the variable and response information, we returned the sample to the population of 9,950.

To reconstruct the second rating session, we drew another random sample of 100 pairs requiring four *categories* (*C*=4) while the other two variables, *difficulty* and *skew*, remained *d_f_*=0 and *s_k_=*.99. Again, we returned each pair back to the population after recording the needed information. We then reconstructed the third session, then the fourth, and so on. We repeated the process for every combination of *category*, *difficulty*, and *skew*, producing 4*8*3=96 sessions.

A few cases can significantly affect π, κ and α when distribution is skewed (9–11,17,50,51,54,55,68,92). To assure stable effects, we resampled three more times to quadruple the number of sessions, so *N_c_*=96*4=384, which was the total number of the reconstructed rating sessions that constituted the “subjects” for this experiment. Each skew condition had an equal number of high- and low- prevalence sessions, that is, each skew=.99 condition had two 1&99 sessions and two 99&1 sessions, and each skew=.75 condition had two 25%75 conditions and two 75&25 sessions.

**II.7. Reconstructed Experiment in Summary.** This was a 4X8X3 between-subject controlled experiment with 4 subjects per cell where each subject was a rating session, as shown in Table 1 in the maintext. The execution took two stages. The first was *individual-level treatment-response*, during which individual-level independent variables, category and difficulty, were manipulated, stimulus and treatment were administered, and individual responses were recorded. The second was *group-level reconstruction*, during which individual responses were sampled and resampled, and the group-level independent variable, skew, was manipulated.

While the treatment and response collection followed the procedure of typical controlled experiment (93), the sampling and resampling benefited from the theories and techniques of bootstrap (94,95); jackknife (96) and Monte Carlo simulation (97).

Simulation is a powerful tool for understanding reliability. But simulations do not measure behavior. They presume certain behaviors then examine their consequences (3,4,9–11,98). A typical individual-level experiment is unsuitable because reliability indices are meaningful only for rating sessions. A session-level experiment would require hundreds of rating sessions, which would be too costly and too difficult to administer. Each rating session would require a fixed level for each independent variable, e.g., all tasks are extremely difficult, have eight categories, and 99% are left, which would deviate too much from realistic rating. Reconstructed experiment offers a useful and feasible addition to our toolkit, allowing observed rater behaviors to be factored into the debate over how raters behave.

1. **Variable Measurements and Calculations**

**III.1. Calculating Chance Agreement (*o_ac_*).** The raters reported few agreements on short bars (0.45%, Table 2 in the maintext), confirming that the main competition was successfully limited between the long bars. It also simplifies the calculation for chance agreement. Assuming no deliberate and systematic errors, each *erroneous agreement* (*o_ae_*), the agreement between two raters choosing a same wrong bar, is considered random. Because there were only two real choices, the probability theory predicates an equal number of agreements falling on the longest bars, thus being correct by chance. Therefore, *observed chance agreement* (*o_ac_*) was calculated by doubling the directly observed erroneous agreement *o_ae_*:

| $o_{ac}=2*o_{ae}$ | ( | 3 | ) |
| --- | --- | --- | --- |

To be sure, we derived another formula for *o_ac_* assuming that sometimes raters had four, six, or eight real choices, as described in Section III.2 below. The two measures yielded essentially the same results. As Eq. 3 is simpler and easier to trace back to the directly observed *o_ae_*, we report statistics based on Eq. 3.

**III.2. Alternative Calculation of Observed Chance Agreement (*o_ac_*).** We identified two formulas for calculating the observed chance agreement (*o_ac_*). The findings section of the manuscript reports the results based on the simpler formula (Eq. 3). All analyses involving *o_ac_* were performed twice using the two different formulas, which produced essentially the same results. We describe the alternative formula (Eq. 4) below.

Some agreements are right, some are erroneous. This study directly observed erroneous agreement (*o_ae_*). As we assume no systematic error, all *o_ae_* are assumed to have come from chance rating, which constitutes the first part of the chance agreement to be estimated.

The observed right agreement (*o_ar_*) includes randomly and systematically right agreement. We need to estimate the former. Due to our design of two long bars and several (0, 2, 4, 6) short bars, the chance agreement came from two types of random selection: between two long bars, and among all bars. When the latter results in an agreement on the longest bar, we call it *right agreement from random choices among all bars* (*a_ra_*)

All agreement on the short bars resulted from raters choosing randomly among all bars. With *C* categories, 1/*C* of such random choices should fall on each bar, including the longest bar. Suppose there are four bars (*C*=4), and *o_s4_* represents observed agreement on the two short bars, the right agreement (on the longest bar) from choosing randomly among four bars equals the agreement on each short bar, which is *o_s4_*/2. Similarly, the right agreement from choosing randomly among six or eight bars is *o_s6_*/4 or *o_s8_*/6, respectively. So the total amount of right agreement from random selection among all bars is *a_ra_*=(*o_s4_*/2)+(*o_s6_*/4)+(*o_s8_*/6), which constitutes the second part of the chance agreement we want to estimate.

Of all observed agreements on the second longest bar (*o_a2_*), some came from random selection among all bars (*a_ra_*), and the rest (*o_a2_*-*a_ra_*) came from random selection between the two long bars. The same amount (*o_a2_*-*a_ra_*) should fall on the longest bar, which constitutes the last part of the chance agreement we want to estimate.

Adding up the three parts, the observed chance agreement *o_ac_* is:

| $o_{ac}=o_{ae}+a_{ra}+{(o}_{a2}-a_{ra})=o_{ae}+o_{a2}$ | ( | 4 | ) |
| --- | --- | --- | --- |

As mentioned, the two approaches of calculating *o_ac_* produced very small differences in means and even smaller differences in correlations. The two formulas therefore corroborate each other.

**III.3. Calculating Observed Reliability (*o_ri_*).** Observed reliability (*o_ri_*) is observed agreement (*a_o_*) minus observed chance agreement (*o_ac_*):

| $\boldsymbol{o}_{\boldsymbol{ri}}\mathbf{=}\boldsymbol{a}_{\boldsymbol{o}}\boldsymbol{-}\boldsymbol{o}_{\boldsymbol{ac}}$ | ( | 5 | ) |
| --- | --- | --- | --- |

1. **Statistical Indicators**

Typical studies calculate estimators to estimate estimands, the targets of estimations. This study observed estimands to evaluate their estimators. We adopted and adapted common indicators, *mean*, *error*, and *r^2^*, to analyze data from this novel design with novel objectives. To guide our choices, we first review the two functions of interrater reliability as estimators.

**IV.1. Approximating and predictive functions of reliability indices.** Reliability indices serve two functions. One is to compare an instrument with fixed benchmarks, such as 0 for absence of reliability, 0.67 for highly tentative reliability, 0.8 for acceptable reliability, and 1 for perfect reliability (46 p147). This function requires an index to *approximate* true reliability in order to *place* accurate scores on instruments, and we need a proximity measure(s) to assess and analyze indices’ ability to approximate true reliability.

Another function is to compare instruments with each other in order to *differentiate* them. This function requires an index to accurately *predict* true reliability, which means to be highly and positively correlated with its estimation target, so that it almost always gives higher scores to more reliable instruments and lower scores to less reliable instruments. We need a correlational measure(s) to evaluate the indices’ ability to predict true reliability.

If an index always approximates the reliability of every individual session perfectly, it also predicts perfectly. Assuming no perfection, however, the prediction-proximity relation is more complicated. A good predictor is not necessarily a good approximator. For example, if a perfect predictor always overestimates by a constant, it’s still a perfect predictor, because all instruments benefit equally. Conversely, a good approximator is not necessarily a good predictor. While a dreadful approximator gives higher score to worse instruments and lower scores to better instruments, its errors could offset each other to make it a perfect approximator on average. Therefore, both proximity and prediction measures are needed.

**IV.2. Proximity Measure I -- Error of Mean (*e_m_*)**. An intuitive proximity measure is *error of mean* (*e_m_*), defined as the difference between the grand average (*mean*) of estimations (*r_i_* or *a_c_*) and the grand average (*mean*) of estimation targets (*o_ri_* and *o_ac_*) . For any reliability index *r_i_* and chance estimator *a_c_*, the error of mean (*e_m_*) calculations are shown as Eqs. 6 and 7.

| $\boldsymbol{e}_{\boldsymbol{m}}\mathbf{(}\boldsymbol{r}_{\boldsymbol{i}}\mathbf{)=}\mathbf{mean}\mathbf{(}\boldsymbol{r}_{\boldsymbol{i}}\boldsymbol{)-}\mathbf{mean}{\boldsymbol{(}\boldsymbol{o}}_{\boldsymbol{ri}}\boldsymbol{)}{-1\leq e}_{m}(r_{i})\leq1$ | ( | 6 | ) |
| --- | --- | --- | --- |
| $\boldsymbol{e}_{\boldsymbol{m}}\mathbf{(}\boldsymbol{a}_{\boldsymbol{c}}\boldsymbol{)}\mathbf{=}\mathbf{mean}\mathbf{(}\boldsymbol{a}_{\boldsymbol{c}}\boldsymbol{)-}\mathbf{mean}{\boldsymbol{(}\boldsymbol{o}}_{\boldsymbol{ac}}\boldsymbol{)}{-1\leq e}_{m}(a_{c})\leq1$ | ( | 7 | ) |

For example, the difference (*e_m_*(*r_i_*)) between κ estimation (*r_i_*) and observed reliability (*o_ri_*), averaged across 384 sessions, would indicate one aspect of κ’s inaccuracy.

As a vector, a positive *e_m_* indicates overestimation, while a negative *e_m_* indicates underestimation. A near zero *e_m_*, however, does not necessarily indicate accuracy for individual rating sessions. Overestimations and underestimations of individual sessions may offset each other to create a small *e_m_*, a phenomenon known as *aggregation bias* or *ecological fallacy* (99,100).

In typical research, however, overestimation of one study does not offset the underestimation of another study. Errors of all directions accumulate or even multiply in terms of social impact. We need an additional measure, which is described below.

**IV.3. Proximity Measure II -- Mean of Errors (*m_e_*).** To avoid aggregation bias, we took the absolute value of the estimation error of each session, |*r_i_*-*o_ri_*| and |*a_c_*-*o_ac_*|, and averaged them across all 384 sessions. The results are *mean of errors* (*m_e_*) for reliability and chance estimations for reliability (*r_i_*) and chance errors (*a_c_*), as shown in Eqs. 8 & 9:

| $\boldsymbol{m}_{\boldsymbol{e}}\left( \boldsymbol{r}_{\boldsymbol{i}} \right)\mathbf{=}\mathbf{mean}\left( \left\vert\boldsymbol{r}_{\boldsymbol{i}}\boldsymbol{-}\boldsymbol{o}_{\boldsymbol{ri}} \right\vert\right) 0\leq m_{e}\left( r_{i} \right)\leq1$ | ( | 8 | ) |
| --- | --- | --- | --- |
| $\boldsymbol{m}_{\boldsymbol{e}}\left( \boldsymbol{a}_{\boldsymbol{c}} \right)\mathbf{=}\mathbf{mean}\left( \left\vert\boldsymbol{a}_{\boldsymbol{c}}\boldsymbol{-}\boldsymbol{o}_{\boldsymbol{ac}} \right\vert\right) 0\leq m_{e}\left( a_{c} \right)\leq1$ | ( | 9 | ) |

Smaller *m_e_* indicates a smaller error hence a better estimator. As a scalar, however, *m_e_* does not differentiate overestimations from underestimations, which vector *e_m_* does.

The spreads of our main variables varied significantly (Lines 4,5,10 &11 of Table 3 in the maintext), which presents another concern. A narrower spread makes *e_m_* and *m_e_* look closer to zero because their baselines (-1~1 or 0~1) do not change with spreads, producing a statistical version of *baseline bias* (101) or *scale of reference bias* (102).

**IV.4. Predictive Accuracy and Share of Influence -- Directional *r*^2^ (*dr^2^*).** As a ratio of regression prediction over total variance, *r^2^* is commonly used to measure *predictive accuracy* (103–105). As a percent of dependent variance explained by independent variable(s), *r^2^* also indicates *share of influence* (103,104,106). As a scalar, however, *r^2^* does not signal direction, while direction is important for this study. There are conflicting expectations about how difficulty or skew affects chance agreement, for example. We added the sign of *r* to *r^2^* to produce a *directional r squared* (*dr*^2^):

| $\boldsymbol{d}\boldsymbol{r}^{\boldsymbol{2}}\boldsymbol{=r*}\left\vert\boldsymbol{r} \right\vert\boldsymbol{-1\leq d}\boldsymbol{r}^{\boldsymbol{2}}\boldsymbol{\leq1}$ | ( | 10 | ) |
| --- | --- | --- | --- |

We use *dr^2^* as the main indicator of indices’ predictive accuracy and various variables’ share of influence.

**IV.5. Regression vs ANOVA.** Experimenters often employ ANOVA for analyzing data. The independent variables of this experiment are on ratio scales, which can be more efficiently analyzed with regression. As regression and ANOVA are mathematically equivalent, there is no loss in essential information or accuracy.

1. **Benchmarks and Thresholds**

This is the first time interrater reliability estimators and their chance agreement estimators are evaluated against their respective estimands, the observed true reliability and observed true chance agreement. No preestablished benchmarks or thresholds are available. Before reporting the outcome, this section lays out the principles that guide the evaluation. Besides helping the reviewers to evaluate our evaluation, we also hope that explicating the principles, if published, may start a conversation about what criteria and principles are appropriate for this type of evaluations.

**V.1. Ideal index outperforms all others.** An ideal index outperforms all other indices on all indicators, producing the largest *dr*^2^ and smallest *m_e_* and *e_m_* for both reliability and chance estimations. Since no such index emerged, the following principles applied.

**V.2. Reliability over chance agreement.** While chance estimation is important for understanding an index’s inside, an index’s value is ultimately judged by the accuracy of its reliability estimation.

**V.3. Prediction (*dr*^2^) over approximation** **(*m_e_* & *e_m_*)**. As said, a good predictor usually gives more reliable instruments higher scores, and less reliable instruments lower scores. A good predictor can be a poor approximator only when its estimations deviate from the true reliability by a near constant across all studies. If the constant can be estimated, such as in studies like this, researchers can add the constant to the estimations to improve the approximation. If the constant cannot be estimated, researchers may collectively adjust the benchmarks to reduce the impact of the across-the-board miss-approximation.

When a good approximator is a poor predictor, its consequences are more severe and harder to remedy. A poor predictor often gives more reliable instruments lower scores, and less reliable instruments higher scores. A poor predictor can be a good approximator only when its errors on individual studies offset each other to lower the across-study errors. The offsetting through averaging does not remedy the underlying cause of the large estimation errors shown in the low correlation.

If we cannot have both, we would trade approximating precision for differentiating precision. When evaluating reliability indices, therefore, more weights should be placed on *dr*^2^ than *m_e_* or *e_m_*.

**V.4. *m_e_ over e_m_*.** To evaluate the indices’ approximation accuracy, we place more weights on mean of errors (*m_e_*) because it is less influenced by aggregation bias.

**V.5. Primary Requirement.** Some disciplines honor *r_i_*>0.8 as the criterion for acknowledging reliability, and *r_i_*>0.67 for highly tentative acknowledgment (31,46,107). Without more reasonable precedents to following, this study tentatively adopts 0.8 and 0.67 as thresholds for *dr^2^*, *m_e_* and *e_m_*. In accordance with Reasoning VI.3 above, we consider Inequality 11 a primary requirement for accepting an index’s validity, where *dr^2^*_(_*_ori_*_&_*_ri_*_)_ represents directional *r^2^* between observed reliability (*o_ri_*) and an index’s estimated reliability (*r_i_*):

| ${\boldsymbol{d}\boldsymbol{r}^{\boldsymbol{2}}}_{\left( \boldsymbol{o}_{\boldsymbol{ri}}\boldsymbol{\&}\boldsymbol{r}_{\boldsymbol{i}} \right)}\boldsymbol{>}\boldsymbol{0}\boldsymbol{.}\boldsymbol{8}\boldsymbol{-}\boldsymbol{1}\boldsymbol{\leq}\boldsymbol{d}\boldsymbol{r}^{\boldsymbol{2}}\boldsymbol{\leq}\boldsymbol{1}$ | ( | 11 | ) |
| --- | --- | --- | --- |

The stated mission of chance-adjusted indices is to outperform percent agreement (*a_o_*), which requires Inequality 12, where *dr^2^*_(_*_ori_*_&_*_ao_*_)_ represents directional *r^2^* between *o_ri_* and *a_o_*.

| ${\boldsymbol{d}\boldsymbol{r}^{\boldsymbol{2}}}_{\left( \boldsymbol{o}_{\boldsymbol{ri}}{\boldsymbol{\&}\boldsymbol{r}}_{\boldsymbol{i}} \right)}\boldsymbol{\geq}{\boldsymbol{d}\boldsymbol{r}^{\boldsymbol{2}}}_{\left( \boldsymbol{o}_{\boldsymbol{ri}}\boldsymbol{\&}\boldsymbol{a}_{\boldsymbol{o}} \right)}\boldsymbol{-}\boldsymbol{1}\boldsymbol{\leq}\boldsymbol{d}\boldsymbol{r}^{\boldsymbol{2}}\boldsymbol{\leq}\boldsymbol{1}$ | ( | 12 | ) |
| --- | --- | --- | --- |

Inequality 11 applies when *dr^2^*_(_*_ori_*_&_*_ao_*_)_<0.8, otherwise Inequality 12 applies.

**V.6.** **Secondary Requirement.** Inequalities 13 &14 serve as the secondary requirement, where *m_e_* _(_*_ri_*_)_ and *m_e_* _(_*_ao_*_)_ represent respectively approximation errors (*m_e_*) of an index (*r_i_*) and *a_o_*.

| ${\boldsymbol{m}_{\boldsymbol{e}}}_{\left( \boldsymbol{r}_{\boldsymbol{i}} \right)}\boldsymbol{<}\boldsymbol{0}\boldsymbol{.}\boldsymbol{2} \boldsymbol{0}\boldsymbol{\leq}\boldsymbol{m}_{\boldsymbol{e}}\boldsymbol{\leq}\boldsymbol{1}$ | ( | 13 | ) |
| --- | --- | --- | --- |
| ${\boldsymbol{m}_{\boldsymbol{e}}}_{\left( \boldsymbol{r}_{\boldsymbol{i}} \right)}\boldsymbol{\leq}{\boldsymbol{m}_{\boldsymbol{e}}}_{\left( \boldsymbol{a}_{\boldsymbol{o}} \right)} \boldsymbol{0}\boldsymbol{\leq}\boldsymbol{m}_{\boldsymbol{e}}\boldsymbol{\leq}\boldsymbol{1}$ | ( | 14 | ) |

Inequality 13 applies when *m_e_* _(_*_ao_*_)_>0.2; Inequality 14 applies otherwise. The threshold 0.2 in Ineq. 13 comes from 1-0.8=0.2, where 0.8 is borrowed from, again, from Krippendorff’s criteria (31,46,107).

**V.7.** **Tentative Requirement.** In case no index meets the primary and secondary requirements, thresholds of 0.67 for *dr^2^* and 0.33 for *m_e_* may be applied for tentative acceptance, again borrowing Krippendorff’s criteria (31,46,107).

**V.8.** **Competitive requirement.** To be among the recommended, an index also needs to outperform all other indices on at least one of the major indicators.

References

1. Chaffee SH. Communication Concept: Explication. Newbury Park, CA: Sage Publications, Inc.; 1991. 96 p.

2. van Swieten JC, Koudstaal PJ, Visser MC, Schouten HJ, van Gijn J. Interobserver agreement for the assessment of handicap in stroke patients. Stroke. 1988;19(5):604–7.

3. Gwet KL. Computing inter-rater reliability and its variance in the presence of high agreement. Br J Math Stat Psychol [Internet]. 2008 [cited 2022 Jan 15];61(1):29–48. Available from: http://doi.wiley.com/10.1348/000711006X126600

4. Gwet KL. Variance estimation of nominal-scale inter-rater reliability with random selection of raters. Psychometrika [Internet]. 2008 [cited 2016 Feb 7];73(3):407–30. Available from: http://link.springer.com/article/10.1007/s11336-007-9054-8

5. Perreault WD, Leigh LE. Reliability of nominal data based on qualitative judgments. J Mark Res. 1989;26(2):135–48.

6. Uebersax JS. The Myth of Chance Corrected Agreement [Internet]. 2009 [cited 2012 Oct 18]. Available from: http://www.john-uebersax.com/stat/kappa2.htm

7. Zhao X, Liu JS, Deng K. Assumptions behind intercoder reliability indices. Ann Int Commun Assoc [Internet]. 2013;36(1):419–80. Available from: http://www.tandfonline.com/doi/abs/10.1080/23808985.2013.11679142?journalCode=rica20

8. Conger AJ. Kappa and Rater Accuracy: Paradigms and Parameters. Educ Psychol Meas [Internet]. 2016 [cited 2022 Jan 15];0013164416663277. Available from: http://epm.sagepub.com/content/early/2016/08/18/0013164416663277.abstract%255Cnhttp://epm.sagepub.com/content/early/2016/08/18/0013164416663277%255Cnhttp://epm.sagepub.com/content/early/2016/08/18/0013164416663277.full.pdf

9. Feng GC. Indexing versus Modeling Intercoder Reliability. Hong Kong Baptist University; 2013.

10. Feng GC. Factors affecting intercoder reliability: A Monte Carlo experiment. Qual Quant [Internet]. 2013 [cited 2022 Jan 15];47(5):2959–82. Available from: http://link.springer.com/article/10.1007/s11135-012-9745-9

11. Feng GC. Underlying determinants driving agreement among coders. Qual Quant. 2013;47(5):2983–97.

12. Grove WM, Andreasen NC, McDonald-Scott P, Keller MB, Shapiro RW. Reliability studies of psychiatric diagnosis: Theory and practice. Arch Gen Psychiatry. 1981;38(4):408–13.

13. Gwet KL. Handbook of Inter-Rater Reliability: The Definitive Guide to Measuring the Extent of Agreement Among Raters. 2nd ed. Gaithersburg, MD: STATAXIS Publishing Company; 2010. 197 p.

14. Kraemer HC. Ramifications of a population model for κ as a coefficient of reliability. Psychometrika [Internet]. 1979 [cited 2022 Jan 15];44(4):461–72. Available from: http://www.scopus.com/scopus/inward/record.url?eid=2-s2.0-0001586045&partnerID=40

15. Spitznagel EL, Helzer JE, John E. Helzer., Helzer JE. A proposed solution to the base rate problem in the kappa statistic. Arch Gen Psychiatry [Internet]. 1985 [cited 2022 Jan 15];42(7):725–8. Available from: http://www.ncbi.nlm.nih.gov/pubmed/4015315

16. Shrout PE, Spitzer RL, Fleiss JL. Quantification of agreement in psychiatric diagnosis revisited. Arch Gen Psychiatry. 1987;44(2):172–7.

17. von Eye A, von Eye M. On the marginal dependency of Cohen’s κ. Eur Psychol. 2008;13(4):305–15.

18. Cohen J. A coefficient of agreement for nominal scales. Educ Psychol Meas [Internet]. 1960 [cited 2022 Jan 15];20(1):37–46. Available from: http://psycnet.apa.org/index.cfm?fa=search.displayRecord&uid=1960-06759-001

19. Hughes MA, Garrett DE. Intercoder reliability estimation approaches in marketing: A generalizability theory framework for quantitative data. J Mark Res [Internet]. 1990 [cited 2022 Jan 15];27(2):185–95. Available from: http://search.ebscohost.com/login.aspx?direct=true&db=buh&AN=9602260627&site=ehost-live

20. Hayes AF, Krippendorff KH. Answering the call for a standard reliability measure for coding data. Commun Methods Meas [Internet]. 2007 [cited 2022 Jan 15];1(1):77–89. Available from: http://www.tandfonline.com/doi/abs/10.1080/19312450709336664

21. Feng GC. Estimating intercoder reliability: a structural equation modeling approach. Qual Quant [Internet]. 2014 Jul 20 [cited 2022 Jan 15];48(4):2355–69. Available from: http://link.springer.com/10.1007/s11135-014-0034-7

22. Feng GC. Intercoder reliability indices: Disuse, misuse, and abuse. Qual Quant [Internet]. 2014 [cited 2022 Jan 15];48(3):1803–15. Available from: http://link.springer.com/article/10.1007/s11135-013-9956-8

23. Feng GC. Mistakes and how to avoid mistakes in using intercoder reliability indices. Methodology [Internet]. 2015 [cited 2022 Jan 15];11(1):13–22. Available from: http://econtent.hogrefe.com/doi/full/10.1027/1614-2241/a000086

24. Benini R. Principii di Demongraphia: Manuali Barbera Di Scienze Giuridiche Sociali e Politiche (No. 29)[Principles of demographics (Barbera Manuals of Jurisprudence and Social Policy)]. Firenze, Italy: G. Barbera; 1901.

25. Bennett EM, Alpert R, Goldstein AC. Communications through limited response questioning. Public Opin Q [Internet]. 1954 [cited 2022 Jan 15];18:303–8. Available from: http://www.ncbi.nlm.nih.gov/entrez/query.fcgi?cmd=Retrieve&db=PubMed&dopt=Citation&list_uids=2189948

26. Goodman LA, Kruskal WH. Measures of association for cross classifications. J Am Stat Assoc. 1954 Dec;49(268):732–64.

27. Guttman L. The test-retest reliability of qualitative data. Psychometrika. 1946;11(2):81–95.

28. Hsu LM, Field R. Interrater agreement measures: Comments on Kappan, Cohen’s Kappa, Scott’s π, and Aickin’s α. Underst Stat. 2003;2(3):205–19.

29. Zwick R. Another look at interrater agreement. Psychol Bull [Internet]. 1988;103(3):374–8. Available from: http://www.scopus.com/inward/record.url?eid=2-s2.0-0024005773&partnerID=tZOtx3y1

30. Cousineau D, Laurencelle L. An unbiased estimate of global interrater agreement. Educ Psychol Meas [Internet]. 2016 [cited 2022 Jan 15];0013164416654740. Available from: http://journals.sagepub.com/doi/abs/10.1177/0013164416654740

31. Krippendorff KH. Reliability in content analysis: Some common misconceptions and recommendations. Hum Commun Res. 2004;30(3):411–33.

32. Scott WA. Reliability of content analysis: The case of nominal coding. Public Opin Q [Internet]. 1955 [cited 2022 Jan 15];19(3):321–325. Available from: http://www.jstor.org/stable/2746450

33. Janson S, Vegelius J. On generalizations of the G index and the Phi coefficient to nominal scales. Multivariate Behav Res. 1979;14(2):255–69.

34. Guilford JP. Preparation of item scores for correlation between individuals in a Q factor analysis. Paper Presented at the Annual Convention of the Society of Multivariate Experimental Psychologists; 1961.

35. Holley JW, Guilford JP. A note on the G-index of agreement. Educ Psychol Meas. 1964;24(4):749–53.

36. Brennan RL, Prediger DJ. Coefficient kappa: Some uses, misuses, and alternatives. Educ Psychol Meas [Internet]. 1981 [cited 2022 Jan 15];41(3):687–99. Available from: http://journals.sagepub.com/doi/10.1177/001316448104100307

37. Byrt T, Bishop J, Carlin JB. Bias, prevalence and kappa. J Clin Epidemiol [Internet]. 1993 [cited 2022 Jan 15];46(5):423–9. Available from: http://www.sciencedirect.com/science/article/pii/089543569390018V

38. Maxwell AE. Coefficients of agreement between observers and their interpretation. Br J Psychiatry. 1977;130(1):79–83.

39. Potter WJ, Levine-Donnerstein D. Rethinking validity and reliability in content analysis. J Appl Commun Res [Internet]. 1999 [cited 2022 Jan 15];27(3):258–84. Available from: http://www.tandfonline.com/doi/abs/10.1080/00909889909365539

40. Gwet KL. Handbook of Inter-Rater Reliability: The Definitive Guide to Measuring the Extent of Agreement Among Multiple Raters [Internet]. 3rd ed. Gaithersburg, MD, USA: Advanced Analytics, LLC; 2012 [cited 2022 Jan 15]. 197 p. Available from: https://books.google.com/books?hl=en&lr=&id=fac9BQAAQBAJ&oi=fnd&pg=PP1&dq=Gwet+K+L&ots=UUdriDAp0a&sig=mKjbb_IW1eNG474Cb0Omp3n5BMk

41. Ji MF, McNeal JU. How chinese children’s commercials differ from those of the united states: A content analysis. J Advert [Internet]. 2001 [cited 2022 Jan 15];30(3):79–92. Available from: http://web.ebscohost.com/ehost/detail?hid=106&sid=c0d4783a-f726-4eea-9dc2-4022b157f163@sessionmgr112&vid=3&bdata=JnNpdGU9ZWhvc3QtbGl2ZQ==#db=buh&AN=5507388

42. Kolbe RH, Burnett MS. Content-analysis research: An examination of applications with directives for improving research reliability and objectivity. J Consum Res. 1991;18(2):243–50.

43. Okazaki S, Rivas JA. A content analysis of multinationals’ Web communication strategies: cross-cultural research framework and pre-testing. Internet Res. 2002;12(5):380–90.

44. Zhao X, Deng K, Feng GC, Zhu L, Chan VKC. Liberal-conservative hierarchies for indices of inter-coder reliability [Internet]. Paper presented at the 62nd annual conference of International Communication Association, Phoenix, Arizona, USA, May; 2012 [cited 2022 Jan 15]. Available from: https://repository.um.edu.mo/handle/10692/102423

45. Krippendorff KH. Estimating the reliability, systematic error and random error of interval data. Educ Psychol Meas [Internet]. 1970 [cited 2022 Jan 15];30(1):61–70. Available from: http://epm.sagepub.com/cgi/doi/10.1177/001316447003000105

46. Krippendorff KH. Content Analysis: An Introduction to its Methodology. Thousand Oaks, CA: Sage; 1980.

47. Uebersax JS. Diversity of decision-making models and the measurement of interrater agreement. Psychol Bull. 1987;101(1):140–6.

48. Andsager JL, Schwartz J. Explicating time: toward making content analysis research describing time frames more meaningful. [Chicago]: Paper Presented at Annual Conference of Association for Education in Journalism and Mass Communication, Chicago; 2012.

49. Bakeman R. Behavioral observation and coding. Handb Res methods Soc Personal Psychol [Internet]. 2000 [cited 2022 Jan 15];138–59. Available from: http://search.ebscohost.com/login.aspx?direct=true&db=psyh&AN=2000-07611-006&site=ehost-live

50. Lombard M, Snyder-Duch J, Bracken CC. Content analysis in mass communication: Assessment and reporting of intercoder reliability. Hum Commun Res [Internet]. 2002 [cited 2022 Jan 15];28(4):587–604. Available from: http://onlinelibrary.wiley.com/doi/10.1111/j.1468-2958.2002.tb00826.x/abstract

51. von Eye A. An alternative to Cohen’s kappa. Vol. 11, European Psychologist. 2006. p. 12–24.

52. Warrens MJ. On marginal dependencies of the 2 × 2 kappa. Adv Stat [Internet]. 2014 [cited 2022 Jan 15];2014:1–6. Available from: http://www.hindawi.com/archive/2014/759527/

53. Aickin M. Maximum likelihood estimation of agreement in the constant predictive probability model, and its relation to Cohen’s kappa. Biometrics [Internet]. 1990 [cited 2022 Jan 15];46:293–302. Available from: http://www.jstor.org/stable/2531434

54. Feinstein AR, Cicchetti D V. High agreement but low Kappa: II. Resolving the paradoxes. J Clin Epidemiol. 1990;43(6):551–8.

55. Feinstein AR, Cicchetti D V. High agreement but low Kappa: I. the problems of two paradoxes. J Clin Epidemiol. 1990;43(6):543–9.

56. Gwet KL. Inter-Rater Reliability: Dependency on Trait Prevalence and Marginal Homogeneity [Internet]. Gaithersburg, MD, USA; 2002 [cited 2022 Jan 15]. Available from: http://hbanaszak.mjr.uw.edu.pl/TempTxt/smirra2.pdf

57. Gwet KL. Handbook of Inter-Rater Reliability: The Definitive Guide to Measuring the Extent of Agreement Among Raters [Internet]. 4th ed. Gaithersburg, MD: Advanced Analytics, LLC; 2014 [cited 2022 Jan 15]. 429 p. Available from: https://books.google.com/books?hl=en&lr=&id=fac9BQAAQBAJ&oi=fnd&pg=PP1&dq=Gwet+K+L&ots=UUdriDAp0a&sig=mKjbb_IW1eNG474Cb0Omp3n5BMk

58. Wongpakaran N, Wongpakaran T, Wedding D, Gwet KL. A comparison of Cohen’s Kappa and Gwet’s AC1 when calculating inter-rater reliability coefficients: a study conducted with personality disorder samples. BMC Med Res Methodol [Internet]. 2013 [cited 2022 Jan 15];13:61. Available from: http://www.pubmedcentral.nih.gov/articlerender.fcgi?artid=3643869&tool=pmcentrez&rendertype=abstract

59. Powers DWM. The problem with Kappa. In: Proceedings of the 13th Conference of the European Chapter of the Association for Computational Linguistics. Association for Computational Linguistics; 2012. p. 345–55.

60. Jeni LA, Cohn JF, De La Torre F. Facing imbalanced data - Recommendations for the use of performance metrics. Proc - 2013 Hum Assoc Conf Affect Comput Intell Interact ACII 2013. 2013;245–51.

61. Bloch DA, Kraemer HC. 2 x 2 Kappa coefficients: Measures of agreement or association. Biometrics. 1989;45(1):269–87.

62. Dewey ME. Coefficients of agreement. Br J Psychiatry. 1983;143(5):487–9.

63. Feuerman M, Miller AR. Relationships between statistical measures of agreement: sensitivity, specificity and kappa. J Eval Clin Pract [Internet]. 2008 [cited 2022 Jan 15];14(5):930–3. Available from: http://onlinelibrary.wiley.com/doi/10.1111/j.1365-2753.2008.00984.x/full

64. Kraemer HC, Bloch DA. Kappa coefficients in epidemiology: An appraisal of a reappraisal. J Clin Epidemiol. 1988;41(10):959–68.

65. Kraemer HC, Periyakoil VS, Noda A. Tutorial in Biostatistics: Kappa coefficients in medical research. Stat Med [Internet]. 2002 [cited 2022 Jan 15];21(14):2109–29. Available from: http://www.ncbi.nlm.nih.gov/pubmed/12111890

66. Roberts C. Modelling patterns of agreement for nominal scales. Stat Med. 2008;27(6):810–30.

67. Williamson JM, Lipsitz SR, Amita K. Manatunga. Modeling kappa for measuring dependent categorical agreement data. Biostatistics [Internet]. 2000 [cited 2022 Jan 15];1(2):191–202. Available from: http://www.ncbi.nlm.nih.gov/pubmed/12933519

68. Vach W. The dependence of Cohen’s kappa on the prevalence does not matter. Vol. 58, Journal of Clinical Epidemiology. 2005. p. 655–61.

69. Rogot E, Irving D. Goldberg. A proposed index for measuring agreement in test-retest studies. J Chronic Dis. 1966;19(9):991–1006.

70. Siegel S, Castellan JNJ. Nonparametric statistics for the behavioural sciences [Internet]. 2nd ed. MacGraw Hill; 1988 [cited 2022 Jan 15]. 213–214 p. Available from: http://scholar.google.com/scholar?hl=en&btnG=Search&q=intitle:Non+parametric+statistics+for+the+behavioural+sciences#9

71. Krippendorff KH. A dissenting view on so-called paradoxes of reliability coefficients. Ann Int Commun Assoc [Internet]. 2013 [cited 2022 Jan 15];36(1):481–99. Available from: http://www.tandfonline.com/doi/pdf/10.1080/23808985.2013.11679143

72. Riffe D, Lacy S, Fico FG. Analyzing Media Messages: Using Quantitative Content Analysis in Research. Mahwah, N J: Lawrence Erlbaum Associates; 1998.

73. Riffe D, Lacy S, Fico FG. Analyzing Media Messages: Using Quantitative Content Analysis in Research [Internet]. 2nd ed. Mahwah, New Jersey and London, New Jersey and London: Lawrence Erlbaum Associates, Publishers; 2005 [cited 2022 Jan 15]. Available from: https://books.google.com.hk/books?hl=en&lr=&id=enCRAgAAQBAJ&oi=fnd&pg=PP1&ots=B00EbKHtj7&sig=e_EdXbsENFS9VfNJR62OrQ00_MM&redir_esc=y#v=onepage&q&f=false

74. Grove WM, Andreasen NC, McDonald-Scott P, Keller MB, Shapiro RW. Reliability studies of psychiatric diagnosis: Theory and practice. Arch Gen Psychiatry. 1981;38(4):408–13.

75. Delgado R, Tibau XA. Why Cohen’s Kappa should be avoided as performance measure in classification. PLoS One [Internet]. 2019 [cited 2022 Jan 15];14(9):1–26. Available from: http://dx.doi.org/10.1371/journal.pone.0222916

76. Feng GC, Zhao X. Do not force agreement – A response to Krippendorff. Methodol Eur J Res Methods Behav Soc Sci [Internet]. 2016 [cited 2022 Jan 15];12(4):145–8. Available from: https://repository.um.edu.mo/handle/10692/26008

77. Zhao X, Feng GC, Liu JS, Deng K. We agreed to measure agreement - Redefining reliability de-justifies Krippendorff’s alpha. China Media Res [Internet]. 2018 [cited 2022 Jan 15];14(2):1. Available from: https://repository.um.edu.mo/handle/10692/25978

78. Zhao X. When to use Scott’s π or Krippendorff’s α, if ever? [Internet]. [St. Louis, USA, August, https://repository.hkbu.edu.hk/coms_conf/3/]: Paper presented at the annual conference of Association for Education in Journalism and Mass Communication; 2011 [cited 2022 Jan 15]. Available from: https://repository.um.edu.mo/handle/10692/102434

79. Zhao X. When to use Cohen’s κ, if ever? [Internet]. [Boston, USA, May, https://repository.hkbu.edu.hk/coms_conf/2/]: Paper presented at the 61st annual conference of International Communication Association.; 2011 [cited 2022 Jan 15]. Available from: https://repository.um.edu.mo/handle/10692/102423

80. Riffe D, Lacy S, Fico FG. Analyzing Media Messages : Using Quantitative Content Analysis in Research. 3rd ed. New York: Routledge; 2014.

81. Krippendorff KH. Misunderstanding reliability. Methodology [Internet]. 2016 [cited 2022 Jan 15];12(4):139–44. Available from: http://econtent.hogrefe.com/doi/full/10.1027/1614-2241/a000119

82. Krippendorff KH. The changing landscape of content analysis: Reflections on social construction of reality and beyond. So CYK, editor. Commun Soc [Internet]. 2019 [cited 2022 Jan 15];47(47):1–27. Available from: https://repository.upenn.edu/asc_papers/604

83. Krippendorff KH. Reliability in content analysis. Hum Commun Res [Internet]. 2004 [cited 2022 Jan 15];30(3):411–33. Available from: http://onlinelibrary.wiley.com/doi/10.1111/j.1468-2958.2004.tb00738.x/abstract

84. Claassen JAHR. The gold standard: not a golden standard. BMJ [Internet]. 2005 [cited 2022 Jan 15];330(7500):1121. Available from: bmj.com

85. Rudd P. In Search of the Gold Standard for Compliance Measurement. Arch Intern Med [Internet]. 1979 Jun 1 [cited 2022 Jan 15];139(6):627–8. Available from: https://jamanetwork.com/journals/jamainternalmedicine/fullarticle/589465

86. Webb PH, Michael L. Ray. Effects of TV Clutter. J Advert Res. 1979;19(3):7–12.

87. Zhao X. Clutter and serial order redefined and retested. J Advert Res [Internet]. 1997 [cited 2022 Jan 15];37(5):57–73. Available from: https://works.bepress.com/xinshu-zhao/11/

88. Jeong Y, Tran H, Zhao X. How much is too much? J Advert Res. 2012;52(1):87–101.

89. Li C. Primacy effect or recency effect? A long-term memory test of super bowl commercials. J Consum Behav. 2010;9(1):32–44.

90. Pieters RGM, Bijmolt THA. Consumer Memory for Television Advertising: A Field Study of Duration, Serial Position, and Competition Effects. J Consum Res. 1997;23(4):362.

91. Terry WS. Serial position effects in recall of television commercials. J Gen Psychol [Internet]. 2005 [cited 2022 Jan 15];132(2):151–63. Available from: http://www.ncbi.nlm.nih.gov/pubmed/15871298

92. Hoehler FK. Bias and prevalence effects on kappa viewed in terms of sensitivity and specificity. J Clin Epidemiol. 2000;53(5):499–503.

93. Montgomery DC. Design and Analysis of Experiments, 7th Edition. John Wiley & Sons. 2009.

94. Efron B. Bootstrap Methods: Another Look at the Jackknife. Ann Stat [Internet]. 1979 [cited 2022 Jan 15];7(1):1–26. Available from: http://projecteuclid.org/euclid.aos/1176344552%5Cnhttps://projecteuclid.org/euclid.aos/1176344552

95. Efron B, Robert J. Tibshirani. An Introduction to the Bootstrap [Internet]. New York and London: Chapman & Hall; 1993 [cited 2022 Jan 15]. 257 p. Available from: http://books.google.com/books?id=gLlpIUxRntoC&pgis=1

96. Shao J, Tu D. The Jackknife and Bootstrap [Internet]. Springer Series in Statistics. New York: Springer Science & Business Media; 1995 [cited 2022 Jan 15]. 516 p. Available from: http://www.loc.gov/catdir/enhancements/fy0815/95015074-d.html

97. Liu JS. Monte Carlo strategies in scientific computing. New York: Springer; 2001.

98. Cicchetti D V., Shoinralter D, Peter J. Tyrer. The effect of number of rating scale categories on levels of interrater reliability: A Monte Carlo investigation. Appl Psychol Meas. 1985;9(1):31–6.

99. Achen CH, Shively WP. Cross-Level Inference. In: Cross-Level Inference. 1995. p. 1–29.

100. Heerink N, Mulatu A, Bulte E, Mulatu A. Income inequality and the environment: Aggregation bias in environmental Kuznets curves. Ecol Econ. 2001;38(3):359–67.

101. Kinsman RA, Staudenmayer H. Baseline levels in muscle relaxation training. Appl Psychophysiol Biofeedback. 1978;3(1):97–104.

102. Groot W. Adaptation and scale of reference bias in self-assessments of quality of life. J Health Econ. 2000;19(3):403–20.

103. Cohen J. Statistical Power Analysis for the Behavioral Sciences [Internet]. 2nd ed. Vol. 2nd. Hillsdale, New Jersey: Erihaum; 1988 [cited 2022 Jan 15]. 567 p. Available from: http://books.google.com/books?id=Tl0N2lRAO9oC&pgis=1

104. Cohen J. A power primer. Psychol Bull [Internet]. 1992 [cited 2022 Jan 15];112(1):155–9. Available from: http://doi.apa.org/getdoi.cfm?doi=10.1037/0033-2909.112.1.155

105. Stigler SM. Francis Galton’s Account of the Invention of Correlation. Stat Sci. 1989;4(2):73–9.

106. Steel RG, James H. Torrie. Principles and Procedures of Statistics. New York: McGraw-Hill; 1960.

107. Krippendorff KH. Content Analysis: An Introduction to its Methodology. 3rd ed. Thousand Oaks, CA: Sage Publications; 2012.
